# *ARABIDOPSIS HOMOLOG OF TRITHORAX1* impacts lateral root development by epigenetic regulation of targets involved in root system architecture

**DOI:** 10.1111/nph.70349

**Published:** 2025-07-07

**Authors:** Selene Napsucialy‐Mendivil, Héctor H. Torres‐Martínez, Gustavo Rodríguez‐Alonso, Diana Marcela Rivera‐Toro, Raúl Alvarez‐Venegas, Marco Adán Júarez‐Verdayes, Svetlana Shishkova, Joseph G. Dubrovsky

**Affiliations:** ^1^ Departamento de Biología Molecular de Plantas, Instituto de Biotecnología Universidad Nacional Autónoma de México (UNAM) Cuernavaca Morelos 62210 Mexico; ^2^ Department of Biology Stanford University Stanford CA 94305 USA; ^3^ Centro de Investigación en Dinámica Celular, Instituto de Investigación en Ciencias Básicas y Aplicadas Universidad Autónoma del Estado de Morelos Cuernavaca Morelos 62210 Mexico; ^4^ Centro de Investigación y de Estudios Avanzados del Instituto Politécnico Nacional, CINVESTAV‐IPN Unidad Irapuato Irapuato Guanajuato 36824 Mexico; ^5^ Departamento de Ciencias Básicas Universidad Autónoma Agraria Antonio Narro Saltillo Coahuila 25315 Mexico

**Keywords:** cell cycle, epigenetic regulation, lateral roots, live imaging, methyltransferase activity, morphogenesis, reactive oxygen species, transcriptomics

## Abstract

- Developmental processes are regulated at multiple levels, including the epigenetic level. Among the epigenetic factors, histone H3 lysine 4 (H3K4) methyltransferases contribute to active transcription of target genes, and here, we explored how the H3K4 methyltransferase ARABIDOPSIS HOMOLOG OF TRITHORAX1 (ATX1) affects *Arabidopsis thaliana* lateral root (LR) primordium (LRP) morphogenesis.
- We examined LR development in a loss‐of‐function null mutant (*atx1‐1*) and a mutant affected in the ATX1 catalytic domain (*atx1setm*) through bright‐field and long‐term time‐lapse confocal microscopy, transcriptomics, and chromatin immunoprecipitation.
- LRP morphogenesis in both mutants was severely abnormal, resulting from altered principal growth directions, and was accompanied by extended cell cycle durations and slower transitions between LRP stages. Among the differentially expressed genes downregulated in *atx1setm*, the most enriched Gene Ontology categories were cell wall organization and H_2_O_2_ metabolism, the latter of which included *PEROXIDASE35* (*PRX35*). The LRP morphogenesis abnormalities were similar in *prx35* and *atx1* mutants. Both the deposition of H3K4me3 at the *PRX35* promoter and the *PRX35* expression in the *atx1setm* mutant were significantly reduced.
- Our results reveal a link between LR development and a redox homeostasis controlled by ATX1 at epigenetic level by maintaining active transcription of *PRX35* and thereby impacting root system formation.

## Resumen

- Los procesos de desarrollo están regulados a diferentes niveles incluyendo el nivel epigenético. La histona metiltransferasa (H3K4me) es un factor epigenético que contribuye a la transcripción activa de sus genes diana. En este trabajo exploramos, como la H3K4 metiltransferasa ARABIDOPSIS HOMOLOG OF TRITHORAX1 (ATX1) afecta la morfogénesis del primordio de raíz lateral, proceso fundamental para que la raíz lateral se desarrolle adecuadamente.
- Usando diferentes enfoques, tales como microscopía de campo claro y confocal, experimentos de rastreo celular en tejidos vivos, análisis del transcriptoma e inmunoprecipitación de la cromatina, estudiamos el desarrollo de la raíz lateral en una mutante nula de pérdida de función (*atx1‐1*) y en una mutante afectada en el dominio catalítico de ATX1 (*atx1setm*), ambas en planta modelo *Arabidopsis thaliana*.
- Encontramos que la morfogénesis del primordio de raíz lateral resultó severamente afectada en las mutantes como consecuencia del cambio en la dirección principal del crecimiento, del ciclo celular alargado y de las transiciones lentas entre los estadios del desarrollo de los primordios. Las categorias de ontología de genes más enriquecidas entre los genes diferencialmente expresados que bajaron su abundancia en la mutante *atx1‐1*, fueron la organización de la pared celular y el metabolismo del peróxido de hidrogeno, dentro de esta última categoría se encontró a la *PEROXIDASE35* (*PRX35*). Las anomalías en la morfogénesis de los primordios de raíz lateral en las mutantes *prx35* fueron similares a las anomalías encontradas en *atx1*. Evidenciamos que en la mutante *atx1setm* tanto la deposición de la marca H3K4me3 en el promotor de *PRX35* como su expresión se disminuyeron significativamente, lo que sugiere que *ATX1* regula a *PRX35* y ambos genes son esenciales para el desarrollo de la raíz en plantas.
- Este trabajo revelo una asociación entre el control del desarrollo de la raíz lateral a nivel epigenético y la homeostasis del estado redox, involucrado en la morfogénesis del primordio de la raíz lateral. Esta asociación impacta el control de la arquitectura del sistema radical en plantas.

## Introduction

Root systems in plants are essential for water and mineral absorption and transport, anchoring the plant in the soil, storing nutrients, and fulfilling other functions (De‐Jesús‐García *et al*., 2020). These systems are formed by producing new roots within parent tissues and by root apical meristem (RAM) activity and rapid cell elongation, which support growth along the root axis. The vast majority of roots in a root system are lateral roots (LRs), which develop from the pericycle of the parent root. Therefore, lateral root initiation (LRI), lateral root primordium (LRP) morphogenesis, and LR emergence are fundamental processes of root system formation.

Regulation of gene expression at the epigenetic level influences all plant development processes, including root development. Posttranslational histone modification has been demonstrated to have an important role in plant development (Takatsuka & Umeda, 2015; Cheng *et al*., 2020). The corepressor complex component *SWIRM domain PAO protein/LSD‐like1* (*SWP1/LDL1*) is a negative regulator of root growth; the *swp1‐1* loss‐of‐function mutant shows increased root growth and LR density (Krichevsky *et al*., 2009; Singh *et al*., 2012). This regulation involves histone deacetylation of chromatin in the root‐specific gene *LATERAL ROOT PRIMORDIUM1* (*LRP1*; Krichevsky *et al*., 2009), which is expressed from the early stages of LRP formation (Smith & Fedoroff, 1995). The expression of genes involved in LRI is either upregulated (*AUXIN RESPONSE FACTOR7* (*ARF7*), *ARF19*, *LATERAL ORGAN BOUNDARIES DOMAIN16* (*LBD16*), and *LBD29*) or downregulated (*AUXIN/INDOLE‐3‐ACETIC ACID14*/*SOLITARY‐ROOT* (*IAA14/SLR*)) in the *swp1‐1* mutant, suggesting that histone deacetylation directly or indirectly affects auxin‐related gene expression in LR founder cells (Singh *et al*., 2012). Importantly, the double mutant affected in *ARF7* and *ARF19* and a single mutant affected in *IAA14/SLR* are deficient in LRI (reviewed in Fukaki *et al*., 2007). PICKLE (PKL; homologous to Chromodomain‐Helicase‐DNA‐Binding ATPase CHD3 (called CHD3) in animals) is involved in chromatin remodeling of *IAA14/SLR* that represses ARF7 and ARF19 activity during LRI in *Arabidopsis thaliana* (Fukaki *et al*., 2006). The PKL protein interacts with RETINOBLASTOMA‐RELATED1 (RBR1), and the PKL‐RBR1 complex negatively regulates LR founder cell divisions via repression of *LBD16* promoter activity (Ötvös *et al*., 2021).

The Polycomb (PcG) and Trithorax (TrxG) group proteins mediate histone modifications and repress or activate gene transcription, respectively (Schuettengruber *et al*., 2011). Many of these proteins are involved in plant development, including root system formation. Polycomb repressive complex 2 (PRC2) catalyzes H3K27 trimethylation (H3K27me3; Bemer & Grossniklaus, 2012), an epigenetic modification usually associated with gene repression. For instance, the EMBRYONIC FLOWER2 (EMF2)–CURLY LEAF (CLF) PRC2 complex acts as a negative regulator of root development, affecting both primary root growth and LR development; *CLF* is preferentially expressed in proliferating cells and represses the activity of the auxin efflux transporter PIN1 by depositing H3K27me3 marks on its chromatin, thereby restricting new LRIs (Gu *et al*., 2014). Furthermore, a H3K27me3 demethylase, RELATIVE OF EARLY FLOWERING6 (REF6), promotes LR formation by removing the repressive mark from the chromatin region where *PIN1*, *PIN2*, and *PIN3* are located (Wang *et al*., 2019).

Notably, TrxG proteins act antagonistically to PcG members; many of them methylate lysine 4 in histone H3 (H3K4), a mark associated with open chromatin states and actively transcribed genes (Alvarez‐Venegas, 2010). The first identified TrxG group histone methyltransferase, ARABIDOPSIS HOMOLOG OF TRITHORAX1 (ATX1), performs dual functions and acts as a facilitator of preinitiation complex assembly and as a H3K4 trimethyl transferase essential for elongation of transcription (Ding *et al*., 2012). It was shown to be required for flower development, as modified organ identity and development of stamenoid petals and carpeloid stamens were reported for the *atx1‐1* mutant (Alvarez‐Venegas *et al*., 2003; Avramova, 2009). In roots, genes encoding H3K4 methyltransferases, *SET DOMAIN GROUP2* (*SDG2*; Yao *et al*., 2013) and *ATX1* (Napsucialy‐Mendivil *et al*., 2014), are also involved in the control of growth and developmental processes. Loss‐of‐function mutants in either of these genes are negatively affected in RAM stem cell function, cell production, and LR formation. The *atx1‐1* mutant has normal LRI but is severely affected in LRP morphogenesis and LR emergence; the LRPs develop slowly, are frequently asymmetric, and sometimes have two domes (Napsucialy‐Mendivil *et al*., 2014). Similar to the delayed emergence of LRs in the *atx1‐1* mutant, the *sdg2‐3* mutant has fewer LRs (Yao *et al*., 2013). It is unknown which specific LRP morphogenesis processes are regulated at the epigenetic level.

Here, we further characterized mutants affected in ATX1 function, including a mutant with a catalytically inactive SET domain, which is involved in H3K4 methylation (*atx1setm*; Ding *et al*., 2012), whose root development was not previously studied. We used live imaging to reveal details of LRP morphogenesis abnormalities. We also performed transcriptomic analysis of the mutant and identified genes potentially regulated by ATX1. Mutations in one of these genes, involved in reactive oxygen species (ROS) homeostasis, resulted in a phenotype similar to that of *atx1* mutants. This work identifies a clear link between LRP morphogenesis and regulation of plant development at the epigenetic level.

## Materials and Methods

### Plant materials and growth conditions

*Arabidopsis thaliana* (L.) Heynh accession Wassilewskija (Ws) or Columbia‐0 (Col‐0) was used as the wild‐type (WT). The mutant alleles used in this study included *atx1‐1* (Alvarez‐Venegas *et al*., 2003) and *atx1;ATX1setm* (Ding *et al*., 2012; hereafter called *atx1setm* for simplicity), both in the Ws background, and the T‐DNA insertion mutants *prx35‐1* (SALK_119795C) and *prx35‐2* (SALK_129866) in the Col‐0 background, obtained from the Nottingham Arabidopsis Stock Centre. The ATX1 overexpression line (ATX1OE) in the Col‐0 background was donated by Z. Avramova. The *PRX35* (AT3G49960) gene name is synonymous with *PER35*. The *prx35* T‐DNA‐insertion mutants were selected by PCR genotyping using primers listed in Supporting Information Table S1. *PEROXIDASE35* (*PRX35*) mRNA abundance was quantified in the *prx35* mutants by reverse transcription quantitative polymerase chain reaction (RT‐qPCR). Seed sterilization and the Murashige and Skoog (MS) medium preparation conditions were described previously (Napsucialy‐Mendivil *et al*., 2014). In all cases, 0.2× MS medium supplemented with 1% sucrose was used. Plants for all experiments were grown under the same conditions at 21°C, under a 16 h : 8 h, light : dark photoperiod with a light intensity of 105 μmol photons m^−2^ s^−1^.

### Plasmid construction and transformation

To study LRP morphogenesis, we aimed to create a marker line that contained a protein expressed in nuclei and plasma membrane. For a nuclear marker, we used a minimal promoter of *TRANSLATIONALLY CONTROLLED TUMOR PROTEIN1* (*TCTP1*) expressed in dividing and elongating cells (Berkowitz *et al*., 2008) and three copies of the mVenus reporter sequence (*pTCTP1::3x mVENUS*); for the plasma membrane marker in the root, we used *mRFP::Wave131* (NPSN12, Geldner *et al*., 2009). To create a marker line for time‐lapse experiments, we generated a *pk7pTCTP1::3xVENUS‐pUB10mRFP‐Wave131* plasmid. First, the FspI and SnaBi fragments of pUB10‐mCherry‐At1g48240/NPSN12 from pNIGEL17‐mCherry were cloned into pJet1.2. The pJet 1.2‐Wave fragment was amplified with 03Wave 5782‐Fw/UBQ10‐Rv primers from the resulting plasmid. The pJet1.2‐Wave‐GW plasmid was created using this fragment, and the pH7WG2D Gateway vector linearized with *Eco*RV. Second, the 3xVENUS fragment from the HBp352 plasmid was amplified with FNLSVENUS/RE9ter primers and inserted into the pJet1.2 plasmid. The *TCTP1* promoter was amplified from Col‐0 genomic DNA with *AtTCTP*‐Fw/*AtTCTP*‐Rv primers and cloned into pJet1.2 (3xVENUS). Both resulting plasmids were cut with SwaI and PstI; then, the three purified fragments of interest were ligated. Finally, mRFP fragment from pENTRDmRFP was ligated into the resulting plasmid. We transformed the WT (Ws) and *atx1‐1* mutant with pk7 plasmid containing *pTCTP13VENUS* and *pUB10mRFP‐Wave* and have found that root growth of the transformed plants was normal. Strong expression of *pTCTP1::3xVENUS* marker was observed in all the nuclei. All primer sequences are provided in Table S1. The Ws and *atx1‐1* mutant (in the Ws background) plants were transformed using *Agrobacterium tumefaciens* GV3101 bearing the respective plasmid using the floral dip method (Clough & Bent, 1998). Transgenic plants were selected on media supplemented with 50 mg l^−1^ kanamycin, and the T4 generation was used for time‐lapse experiments. For unknown reasons, we did not detect the expression of the *mRFP‐WAVE* marker in the plasma membrane. The respective lines were called Ws *TCTP1::3xVENUS* and *atx1‐1 TCTP1::3xVENUS* and used for time‐lapse analysis.

### Microscopy and time‐lapse experiments

Roots were cleared with an acidified methanol procedure (Malamy & Benfey, 1997) with modifications as described (Dubrovsky *et al*., 2006), and whole‐mount preparations were analyzed using an Olympus BX53 microscope (Olympus, Tokyo, Japan) equipped with differential interferential contrast optics (Nomarski).

Confocal laser scanning microscopy and time‐lapse experiments to analyze LRP morphogenesis from Stage I onwards were conducted using an inverted confocal laser scanning microscope setup as described (Reyes‐Hernández *et al*., 2019). Briefly, a confocal system was constructed around a Zeiss Axiovert 200 M microscope (Oberkochen, Germany), featuring a high‐speed galvo‐resonant scanner (SCAN‐VIS), a 488‐nm laser source, and a filter cube equipped with 525/45‐ and 630/92‐nm bandpass filters for green and red fluorescent proteins, respectively. The system also included a dual‐channel PMT module, a linear motor travel XY stage, and a Z‐axis piezo stage with controllers (all sourced from Thorlabs Inc., Newton, NJ, USA). Imaging was performed using a Zeiss C‐Plan NEOFLUAR ×40, 0.75NA objective (Oberkochen, Germany).

For time‐lapse experiments, seedlings at 5 d after germination (dag) were carefully positioned in a one‐well Thermo Scientific™ Nunc™ Lab‐Tek™ II Chamber Slide™ System with a #1.5 cover glass at the base of the chamber (Thermo Fisher Scientific, Waltham, MA, USA). The root system was covered with a rectangular piece (*c*. 3 mm thick) of 0.2× MS medium with 1% sucrose and 0.8% agar. To acclimate the seedlings to their new environment, they were horizontally maintained within the growth chamber for 1 h before the beginning of the experiments. Subsequently, the Chamber Slide™ System was placed on a motorized microscope stage. The shoots in the chamber were illuminated using an FP600ERT, a 600‐μm core multimode fiber connected to a fiber‐coupled LED MWWHF2 light source, with a color temperature of 4000 K and an intensity of 16.3 mW (Thorlabs Inc.). To ensure a 16‐h photoperiod, the light source was controlled through a timer. The temperature in the room was kept at 21°C throughout the experiment, providing an optimal and controlled environment for root imaging studies.

An image processing pipeline was implemented to optimize the signal‐to‐noise ratio. Initially, the parallel iterative deconvolution plug‐in (Schindelin *et al*., 2012) was applied, utilizing a configured theoretical Point Spread Function obtained from https://www.optinav.info/imagej.html. Images were further refined using the Gaussian Blur 3D tool (Schindelin *et al*., 2012). Starting from Stage I LRP, nuclei of all cells and their progenies were tracked over time using the Fiji plug‐in LiPlaCeT tools (Schindelin *et al*., 2015; Rueden *et al*., 2017; Hernández‐Herrera *et al*., 2022). The tracked data were manually curated. Visualization Toolkit (*.vtk) files, compatible with ParaView, were created. These files contained information on the position of tracked cells in real coordinates (X, Y, and Z) for each time point. With this application, color‐coded clones of a cell's progeny were viewed in 3D, rotated, and analyzed, and a colormap was used to visualize each cell lineage. From these data, cell cycle durations and principal growth directions (PGDs) were extracted as described (Torres‐Martínez *et al*., 2020; Hernández‐Herrera *et al*., 2022).

### RNA extraction, RT‐qPCR, and transcriptome analysis

Total RNA was extracted from roots of WT and *atx1setm* seedlings 8–10 dag using TRIzol reagent (Invitrogen/Thermo Fisher Scientific, Waltham, MA, USA), according to the manufacturer's instructions. RT‐qPCR analysis was performed using a Light Cycler (R) Nano Real‐Time PCR System (manufactured under license from IT‐IS Life Science Ltd), with SYBR Green (Thermo Fischer Scientific) as a fluorescent probe. First‐strand cDNA synthesis was performed using Superscript II Reverse Transcriptase (Thermo Fischer Scientific) and oligo‐dT primer. For RT‐qPCR reactions, 75 ng of cDNA per reaction was used. The qPCR primers used are listed in Table S1. Data were normalized to the expression of two reference genes: *UBQ10* (At4g05320) and *EF1α* (AT1G07940; Czechowski *et al*., 2005), and the expression levels were calculated according to Vandesompele *et al*. (2002). Three biological replicates with two technical replicates each were performed. RNA‐Seq was carried out on the *atx1setm* and WT roots of 8 dag plants (two biological replicates). Library preparation and 50 cycles of single‐end sequencing on the HiSeq 2000 (Illumina) platform were performed at BGI‐Tech, Hong Kong. The quality of the sequence reads was analyzed with CLC Genomics Workbench v7.5 (CLC GW, CLC bio; Qiagen, Hilden, Germany; http://www.clcbio.com/) (Mortazavi *et al*., 2008). The reads were mapped against the *Arabidopsis* reference genome (TAIR10). For differential expression analysis, we used the extraction of differential gene expression test with fold change ≥ 3, *P*‐value ≤ 0.0001, and false discovery rate‐corrected *P*‐value ≤ 0.01 as parameters. To visualize transcript abundance as heatmaps, the reads per kilobase per million mapped reads values of each sample were mean‐centered and scaled by the SD to obtain the *z*‐score; the heatmap was obtained with the Pheatmap package in R v.4.1.2. The RNA‐seq data were validated by measuring the relative expression of selected genes by RT‐qPCR (Table S1). All data of *atx1setm* RNA‐seq were deposited in the Gene Expression Omnibus (GEO) of the National Center for Biotechnology Information and are accessible through GEO accession no.: GSE252067.

### Growth analysis

Quantitative analysis of root development was performed as described previously (Napsucialy‐Mendivil *et al*., 2014). For the H_2_O_2_ treatment, 5 dag seedlings were transferred to the same medium or medium supplemented with 2 mM H_2_O_2_ and grown for an additional 3 d. The percentage of abnormal LRPs in 8 dag WT (Col‐0) and *prx35‐2* seedlings treated with 2 mM H_2_O_2_ (Sigma‐Aldrich) was estimated only in the LR formation zone.

### Chromatin immunoprecipitation analysis

Chromatin isolation and immunoprecipitation were performed as described previously (Saleh *et al*., 2008; Martínez‐Aguilar *et al*., 2016), with some modifications. For chromatin immunoprecipitation (ChIP) assays, whole root samples (500 mg) from *c*. 100 Ws and 250 *atx1setm* 8 dag plants were used. The samples were fixed with 1% formaldehyde (v/v), and the chromatin was isolated and digested at 37°C for 15 min with 15 U of micrococcal nuclease (cat. no.: 88216; Thermo Scientific) to generate 250‐bp fragments. The samples were centrifuged at 16430 ***g*** for 10 min at 4°C, and the digested chromatin was collected and frozen at −80°C until it was immunoprecipitated. Aliquots of the digested chromatin (diluted 10‐fold) were used in each immunoprecipitation. We utilized the EZ‐Magna ChIP Kit (cat. no.: 17‐408; Millipore) and the Magna ChIP Protein A + G Magnetic Beads (cat. no.: 16‐663; Millipore), following the manufacturer's instructions. The immunoprecipitation was performed overnight at 4°C, using 5 μl of ChIPAb + Trimethyl‐Histone H3 (Lys4, cat. no.: 17‐614; Millipore). Following immunoprecipitation, the Protein A + G bead‐antibody/chromatin complex was separated with the magnetic Magna Grip Rack separator (cat. no.: 20‐400; Millipore) and washed; the protein–DNA complexes were eluted, crosslinks were reversed to free DNA, and DNA was purified. An aliquot of the initially digested chromatin was used as a template for input samples after its purification. ChIP experiments were performed using two biological replicates with two technical replicates each; all PCR reactions were run in triplicate per sample. PCR amplification was carried out using the Maxima SYBR Green/ROX qPCR Master Mix (Thermo Fisher Scientific Inc., following the manufacturer's instructions) on a C1000 Thermal Cycler (Bio‐Rad Laboratories Inc., Hercules, CA, USA). Primers used are listed in Table S1. ChIP‐qPCR data were normalized to the input sample (1% of input chromatin), according to the percent input method (% Input = 2^((Cq(IN)‐Log^_2_^(DF))‐Cq(IP))^ × 100; Solomon *et al*., 2021).

### Statistical analysis

All experiments were repeated at least two times. Statistical analysis for samples that passed the normality test was performed using a Student's *t*‐test by evaluating the two‐tailed *P*‐value, Brown–Forsythe test, or one‐way ANOVA. When the normality test failed, nonparametric multiple comparison procedures (Holm–Sidak method or Mann–Whitney rank sum test) were applied. The statistical analysis used in each case is indicated in the figure legends. For statistical analysis and graph preparation, SigmaPlot v.12 (Systat Software Inc., Chicago, IL, USA) and OriginPro (OriginLab Corp., Northampton, MA, USA) were used.

## Results

### Histone methyltransferase activity of ATX1 is essential for LR development

**Fig. 1:**
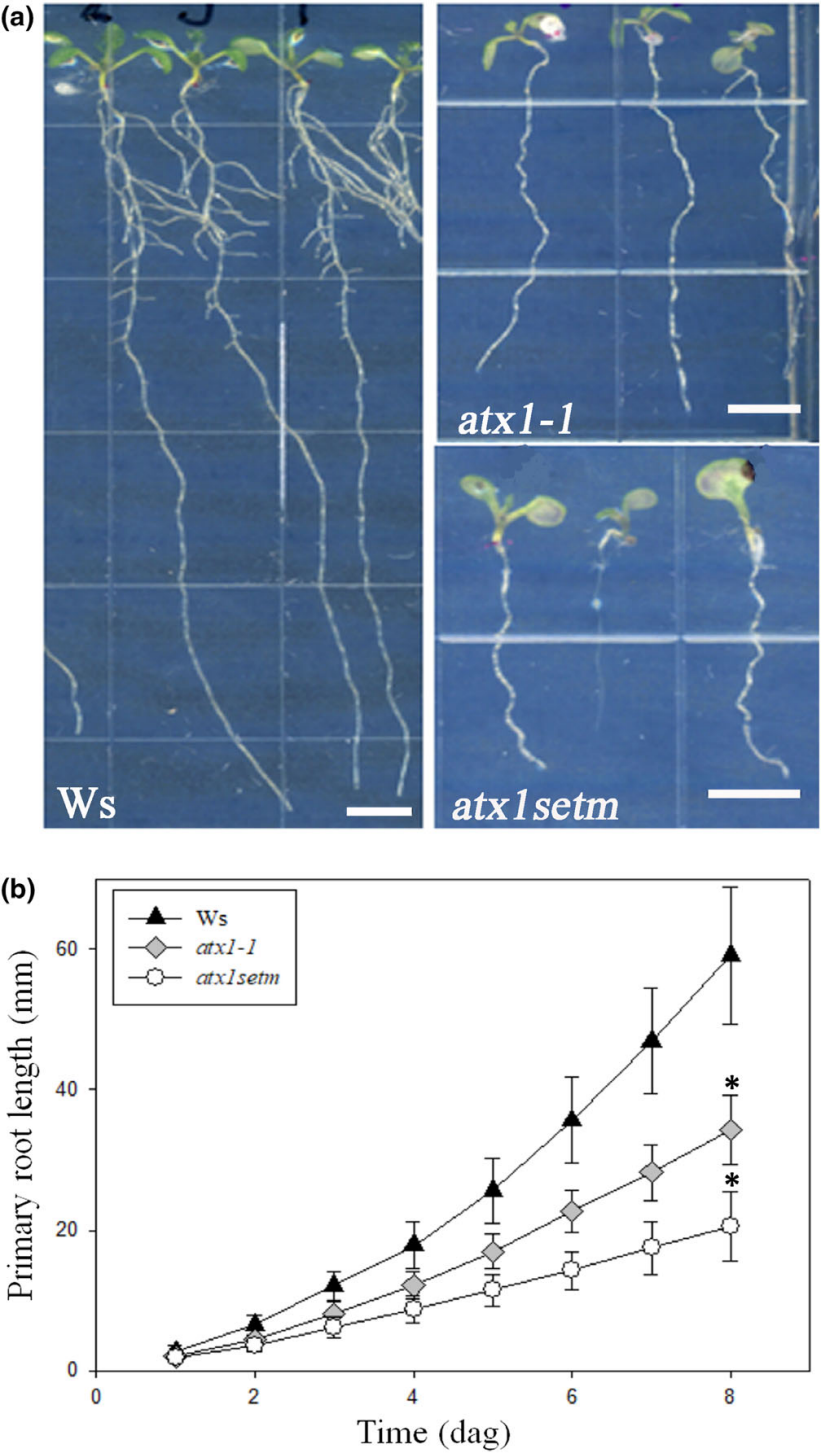
Histone methyltransferase activity of ARABIDOPSIS HOMOLOG OF TRITHORAX1 (ATX1) is essential for *Arabidopsis thaliana* root development. (a) Wild‐type (Wassilewskija (Ws)), *atx1‐1*, and *atx1‐1*; *ATX1‐setm* (*atx1setm*) seedlings at 8 d after germination (dag). Bars, 5 mm. (b) Primary root growth dynamics of Ws, *atx1‐1*, and *atx1setm*. Values are means ± SD (*n* = 25–31). Combined data of three independent experiments are shown; asterisks indicate statistical differences between each of the mutants and the Ws and among the two mutant alleles (*, *P* < 0.001, one‐way ANOVA).

ATX1‐dependent H3K4me3 is required for efficient elongation of transcription, and histone methylation (Ding *et al*., 2012). To compare how root system formation depends on the complete loss of *ATX1* expression vs its development when only methyltransferase activity is compromised, we analyzed root development in the *atx1‐1* null mutant (Fig. S1a) and *atx1setm*, which possesses catalytically inactive ATX1 harboring five tyrosine‐to‐alanine substitutions in the SET domain in the *atx1‐1* background (Ding *et al*., 2012; Fig. S1b). Therefore, while ATX1 was absent in the *atx1‐1* mutant, the protein was present in the *atx1setm* mutant, but its methyltransferase activity was abolished. Surprisingly, the *atx1setm* seedlings were affected to a greater extent than the *atx1‐1* null mutant, as their primary roots were 65 and 40% shorter than those of the WT (Ws), respectively (Fig. 1a,b). This finding suggests that the ATX1 protein lacking methyltransferase activity prevents H3K4 methylation by other TrxG proteins in the proximity of the ATX1 DNA‐binding sites.

**Fig. 2:**
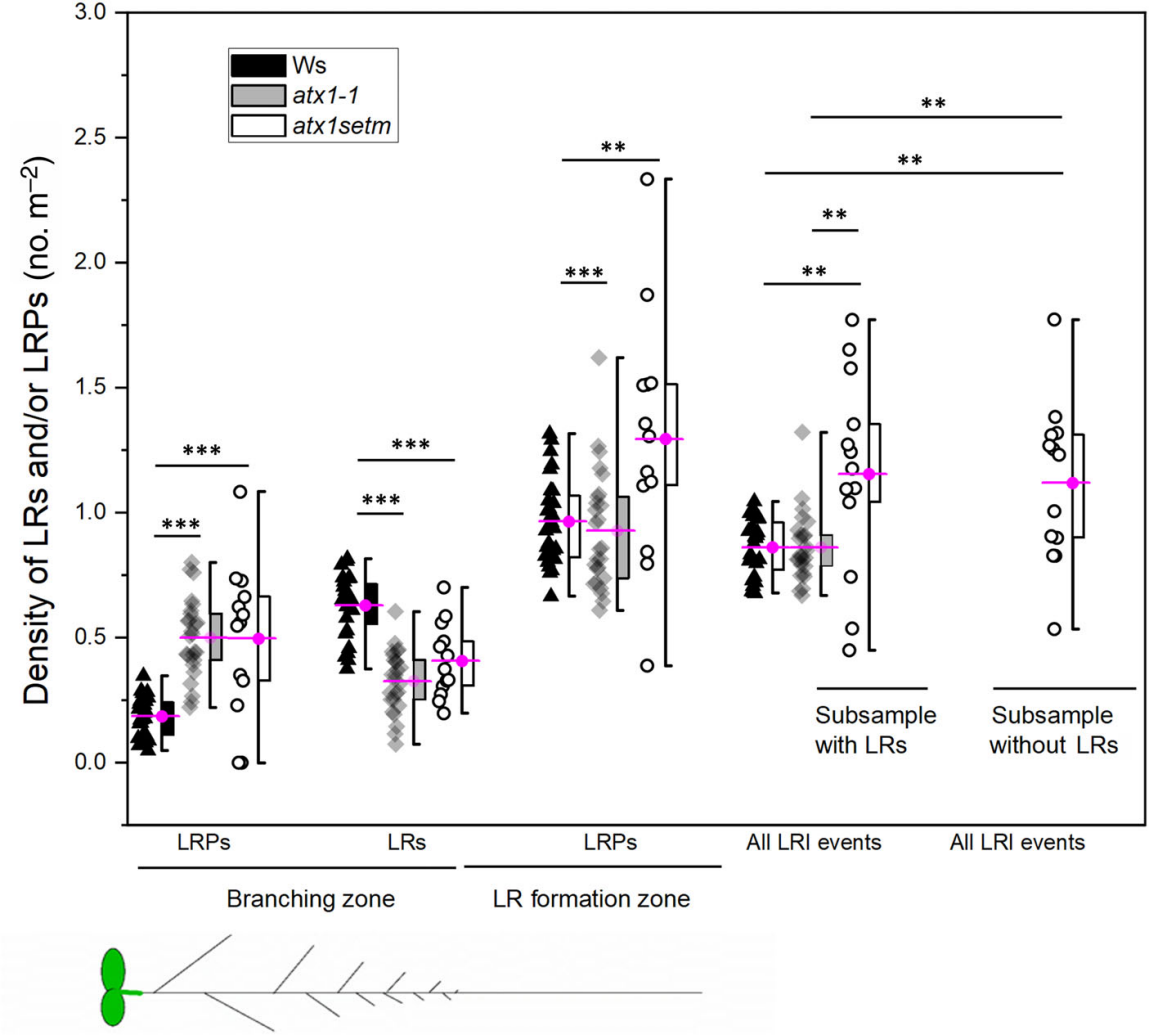
Quantitative analysis of *Arabidopsis thaliana* lateral root (LR) formation in the *atx1‐1* and *atx1setm* mutants in comparison with the wild‐type (Wassilewskija (Ws)) in seedlings 8 d after germination. Lateral root primordia (LRPs) were analyzed within branching and LR formation zones, depicted at the bottom. All lateral root initiation (LRI) events were compared separately for a subsample of *atx1setm* plants that formed LRs and for a subsample in which LRs did not emerge. All other data include *atx1setm* subsample with LRs. Only statistically significant differences are indicated for each parameter using pairwise comparison among different genotypes by the Holm–Sidak method at ***, *P* < 0.001 and **, *P* < 0.01. Mean values (magenta) are shown; boxes span from 25 to 75% percentiles, and whiskers range from a minimum to a maximum value. Combined data of three independent experiments are shown: *n* ranges from 26 to 28 seedlings.

We have previously shown that LR density in the root branching zone (a root portion with emerged LRs, Dubrovsky & Forde, 2012; Fig. 2) in *atx1‐1* was lower than that in the WT, but the LRP density in this zone was greater (Napsucialy‐Mendivil *et al*., 2014). Similar changes were found in the *atx1setm* mutant (Fig. 2), suggesting that LR emergence was delayed. This was in agreement with the finding that a fraction of *atx1setm* seedlings (50%) did not have emerged LRs at all (this phenotype was not present in *atx1‐1* mutant). Despite the higher density of LRPs in *atx1setm* seedlings, LRI was not affected. The density of LRPs in a subsample of seedlings without LRs was the same as the density of all LRI events (including both LRs and LRPs) in a subsample that formed LRs (Fig. 2, compare all LRI events). The density of all LRI events in *atx1‐1* was the same as in the WT, and in *atx1setm*, it was greater than in WT. However, because the fully elongated cortical cells were shorter in the *atx1setm* seedlings (Fig. S2a), the LRI index (i.e. the number of LRI events in a root portion corresponding to 100 cortical cells in a file; Dubrovsky *et al*., 2009) did not differ between the mutants and the WT (Fig. S2b). Therefore, LRI was quantitatively similar to WT in both *atx1* mutants. The finding that in *c*. half of 8 dag *atx1setm* seedlings no LR emerged suggests that the H3K4me3 marks in the ATX1 binding sites are required for normal timing of LRP development in WT plants.

**Fig. 3:**
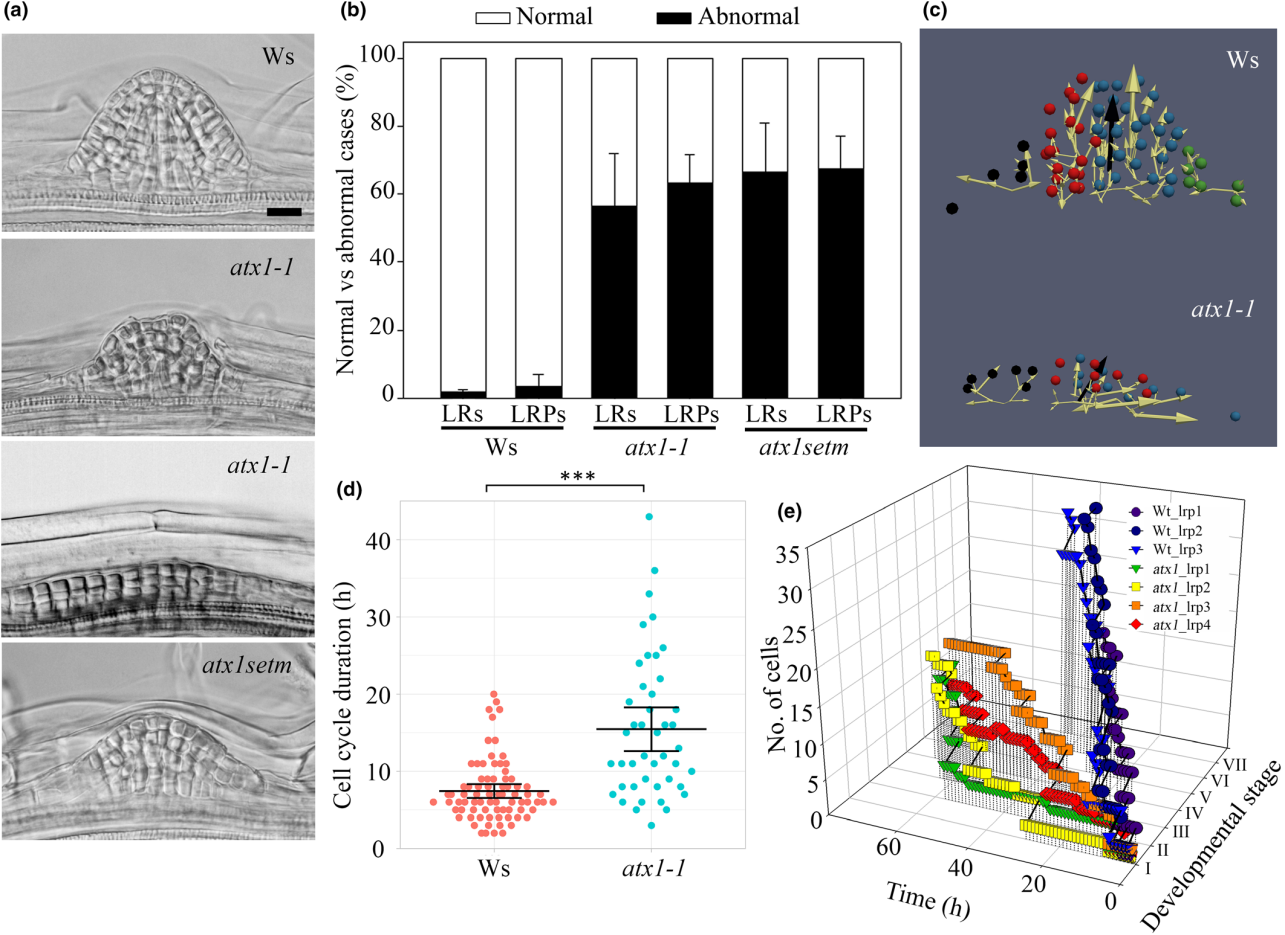
ARABIDOPSIS HOMOLOG OF TRITHORAX1 (ATX1) is required for correct timing during lateral root (LR) primordium (LRP) morphogenesis in *Arabidopsis thaliana*. (a) Representative images of LRPs in the wild type (Wassilewskija (Ws)), *atx1‐1*, and *atx1setm* mutants. Bar, 20 μm; all panels are at the same magnification. (b) Percentage of abnormal LRPs and LRs in Ws, *atx1‐1*, and *atx1setm* seedlings at 8 d after germination. Combined data of three independent experiments; mean ± SD, total *n* range from 26 to 28 seedlings. (c–e) Analysis of cell proliferation dynamics in Ws and *atx1‐1*, both transformed with the *pTCTP1::3x mVENUS* construct, using time‐lapse experiments. (c) Principal growth directions (PGDs) were analyzed in Ws and *atx1‐1* mutant LRPs over a 37‐h period after the time‐lapse experiment started. During this time, depicted LRPs reached Stages VII and III for Ws and *atx1‐1*, respectively. The progeny of each cell was followed from Stage I LRPs, and related color‐coded clones are shown. Yellowish arrows indicate the PGDs of each clone, and black arrows indicate the average PGD of all the clones. Note that the latter in *atx1‐1* is not perpendicular to the parent root growth axis, as in Ws. (d) Analysis of cell cycle duration in the developing LRPs in Ws and the *atx1‐1* mutant. Data show means ± SD. Individual values are shown. A statistical significance (***) is at *P* < 0.001, Mann–Whitney rank sum test; wild‐type *n* = 80 cells from three LRPs, developed from Stages I to VII; each LRP was from different seedlings; in *atx1‐1*, *n* = 46 cells from four LRPs developed from Stages I to V; each LRP was from a different seedling. (e) Analysis of LRP development showing the dynamics of cell number increase at each developmental stage. The data in (d) and (e) were extracted by tracking the progeny of each cell in the central portion of the master cell file from each LRP: *n* = 78 cells from three LRPs in Ws and 44 cells from four LRPs in the *atx1‐1* mutant. Time‐lapse experiments started at Stage I LRPs and spanned 72 h for the mutant; cell tracking for Ws was performed only until Stage VII that ranged between 34 and 40 h from the beginning of the experiment.

Next, we addressed the possible reasons for the delayed or arrested LRP formation in the *atx1‐1* and *atx1setm* mutants. In the *atx1‐1* mutant, LRP morphogenesis is severely affected, and asymmetric one‐dome, extended, or two‐dome LRPs are formed (Napsucialy‐Mendivil *et al*., 2014). Similar abnormalities were observed in the roots of the *atx1setm* mutant (Figs 3a, S3), and their frequency was even greater than in *atx1‐1*: 50% of LRs and 60% of LRPs formed by the *atx1‐1* mutant exhibited abnormalities, whereas 67% of LRs and 67% of LRPs formed by the *atx1setm* mutant exhibited abnormalities (Fig. 3a,b). As expected, the percentage of LRP abnormalities when *ATX1* was overexpressed in *atx1‐1* background was similar to that of WT (Fig. S4).

To investigate the cellular basis of these morphogenetic abnormalities and track the progeny of each cell, we created nuclear marker lines (*pTCTP1::3x mVENUS*) in *atx1‐1* and the WT (Ws). To address possible reasons for a lower density of emerged LRs in the root branching zone of the *atx1‐1* mutants (Fig. 2), we analyzed the distribution of LRP developmental stages by performing long‐term time‐lapse experiments. In *atx1‐1*, the LRP reached Stages II, III, IV, and V (as defined in Malamy & Benfey, 1997) two to three times slower than those in the WT (Fig. 3e; Table 1). During the 72‐h experiment, the LRPs in the *atx1‐1* mutant reached Stage V, while in the WT, Stage VII LRPs were observed by 38 h (Table 1; Fig. S5). To estimate cell cycle duration, starting from Stage I LRP, a progeny of each cell was followed. The Live Plant Cell Tracking (LiPlaCeT) Fiji plug‐in (Hernández‐Herrera *et al*., 2022) was used to record the time when each cell just divided. Each daughter cell was monitored in time‐lapse, and the time span until the next division was considered a duration of the cell cycle. The *pTCTP1::3x mVENUS* nuclear marker (Fig. S5) was used to identify each nucleus and cell progenies. This analysis showed that slow LRP formation and slow transition to each subsequent stage in the *atx1‐1* mutant were caused largely by extended cell cycle duration (Fig. 3d). In *atx1‐1* LRPs, the cell cycle was 2.1 times longer than that in the WT (Average of 7.44 h for WT and 15.45 h for *atx1‐1*).

**Table 1:** Timing of lateral root primordium (LRP) formation in mutant and wild‐type seedlings of *Arabidopsis thaliana*.

Developmental stage	Ws	*atx1‐1*	Statistical analysis, *P*
II	6 ± 2.0	13.5 ± 11.0	0.114^a^
III	14.3 ± 4.0	41.3 ± 15.6	0.0356
IV	17.7 ± 4.2	56.3 ± 10.2	0.002
V	22 ± 3.5	62.3 ± 8.0	0.0002
VI	28.3 ± 3.8	Did not reach the stage during 72 h	
VII	36.7 ± 6.5	Did not reach the stage during 72 h	

The data were extracted from time‐lapse experiments and show the duration in hours from Stage I LRP until the developmental stage indicated. Mean ± SD; *n* = 3 to 4. Two‐tailed Student's *t*‐test *P*‐values are indicated. ^a^Indicates that normality test failed, and a nonparametric Mann–Whitney rank sum test was used.

Furthermore, an analysis of the dynamics of cell number increase at each LRP stage revealed that the slow transitions between stages in the *atx1‐1* mutant (Fig. 3e) were associated with lower cell production. In the *atx1‐1* mutant, the transition from Stages II to III was 3.3‐fold longer than in the WT. As many LRPs in the *atx1‐1* mutant had abnormal shapes (Fig. 3a,b), we also conducted 3D visualization of PGDs obtained from cell tracking of progenies of Stage I LRP located in the master cell file defined by von Wangenheim *et al*. (2016). The PGD is the average vector from all cell division vectors of progenies of each individual cell present at the beginning of the experiment (Hernández‐Herrera *et al*., 2022). This analysis showed that while in the WT, the PGD is properly established during LRP morphogenesis, and it is orthogonal to the longitudinal axis of the parent root, in the *atx1‐1* mutant, this correct PGD fails to establish. The calculated growth direction in *atx1‐1* was intermediate between the longitudinal and transverse direction in relation to the parent root, tending to be tangential (Fig. 3c; Video S1). This explains why it was common to find longitudinally extended LRPs in the mutant (Napsucialy‐Mendivil *et al*., 2014; Fig. 3a).

Our observation that the *atx1setm* mutant exhibited even stronger developmental defects in LR formation than the *atx1‐1* null mutant (Figs 1, 2, 3) suggests that in the null mutant the H3K4me3 marks could be added also by other methyltransferases. In summary, comprehensive analysis of cell proliferation features in time‐lapse experiments revealed that ATX1 activity plays an important role during LRP morphogenesis through its impact on cell cycle duration, cell division orientation, and establishment of a new growth direction.

### Identification of differentially expressed genes related to plant development in the *atx1setm* mutant

**Fig. 4:**
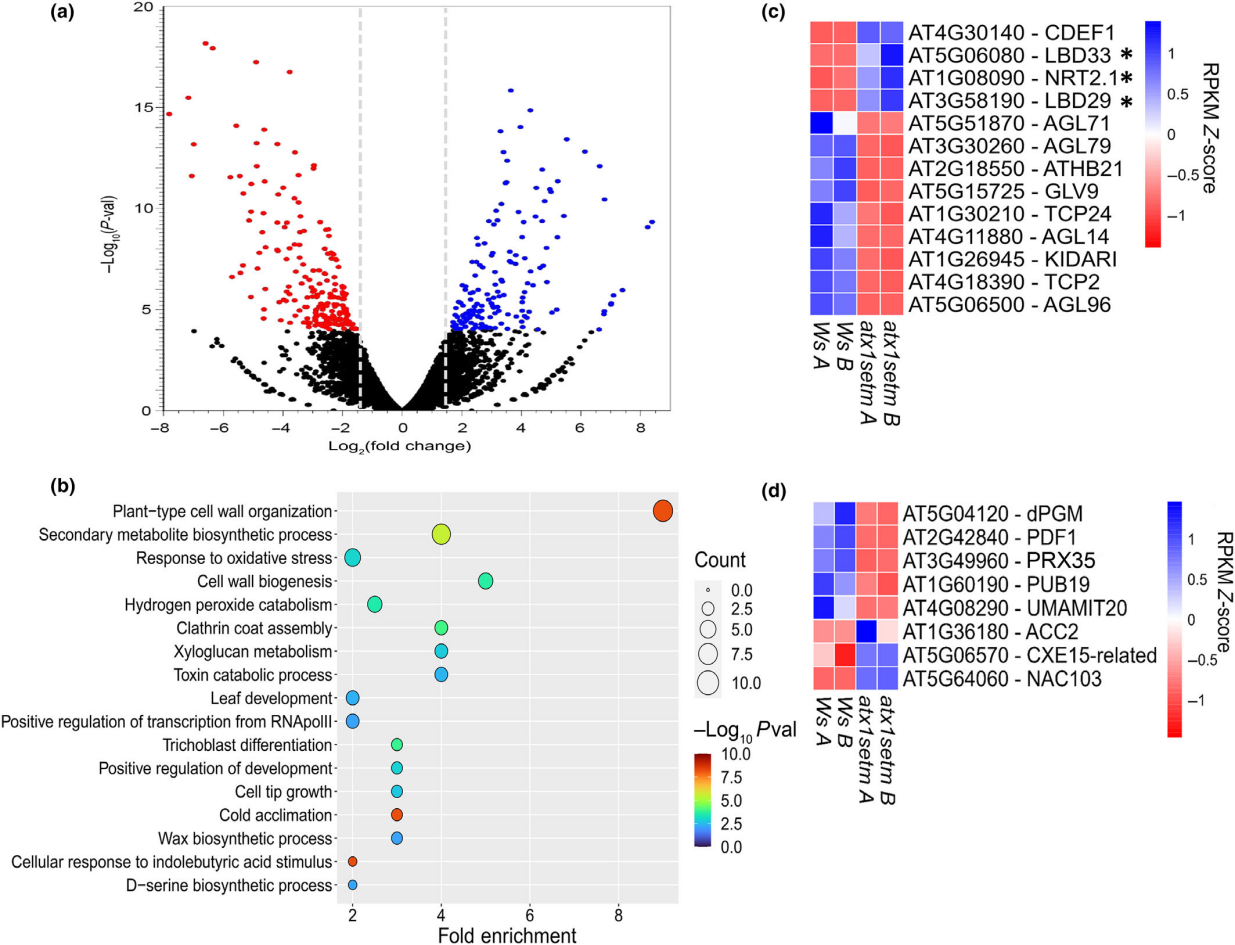
Transcriptomic analysis of the *atx1setm* mutant of *Arabidopsis thaliana*. (a) Volcano plot displaying upregulated (blue) and downregulated (red) genes in the *atx1setm* mutant compared with the wild type (Ws); fold change ≥ 3, *P*‐value ≤ 0.0001. (b) Gene Ontology (GO) enrichment analysis of downregulated genes; circle size is proportional to the number, or count, of occurrences for each biological process and filled with a color scale according to the −Log_10_ *P*‐value of the enrichment, which is given on the *X*‐axis. (c) Reads per kilobase per million mapped reads (RPKM) *Z*‐score of genes relevant for root system architecture. Genes reported as directly related to LRP development are indicated with an asterisk. Each column corresponds to a biological replicate of the indicated genotype. (d) RPKM Z‐score of genes differentially expressed in the *atx1setm* mutant, which were previously reported as being preferentially expressed in the pericycle (Parizot *et al*., 2012).

To investigate the possible target genes of ATX1 related to its methyltransferase activity and linked to root development, we performed RNA‐Seq of the WT and *atx1setm* roots using 8–10 dag seedlings. As in half of all *atx1setm* plants, LRs did not emerge, and in another half the density of LRs in the branching zone was significantly lower than that in WT (Fig. 2), for RNA‐Seq sample the whole roots were collected. Transcriptome profiling showed that 344 genes were differentially expressed (≥ threefold change) in roots of the *atx1setm* mutant. Of these, 189 genes were downregulated, and 155 were upregulated (Fig. 4a; Table S2). To explore the molecular pathways affected in the *atx1setm* mutant, we performed Gene Ontology analysis of differentially expressed genes (DEGs) focusing on biological process (Fig. 4b). As H3K4me3 is a chromatin modification mark associated with active gene transcription (Benayoun *et al*., 2014), to identify putative targets of ATX1, we analyzed the downregulated genes in more detail. In agreement with developmental defects identified in the *atx1setm* mutant, we found significant enrichment of genes important for plant‐type cell wall organization (*P* = 3.26 × 10^−9^), trichoblast differentiation (*P* = 4.61 × 10^−5^), hydrogen peroxide catabolic process (*P* = 3.57 × 10^−4^), cell tip growth (*P* = 3.75 × 10^−4^), and positive regulation of development (*P* = 1.28 × 10^−3^; Fig. 4b). We previously showed that the abnormalities in *atx1‐1* LRP morphogenesis were not directly related to auxin response (Napsucialy‐Mendivil *et al*., 2014). In accordance with our transcriptomic analysis, no genes involved in auxin metabolism, perception or signaling were found among up‐ and downregulated genes, and only three indirectly auxin‐related genes were detected (Fig. S6). Surprisingly, neither cell‐cycle‐related downregulated DEGs were found, although three DEGs were upregulated (Fig. S6).

To validate the obtained RNA‐seq data, we performed RT‐qPCR analysis of selected genes (Table S1). We chose three downregulated genes (*GOLVEN 9* (*GLV9*), *RELATED TO AP2.1* (*RAP2.1*), and *COBRA‐LIKE9* (*COBL9*)) and three upregulated genes (*SIAMESE‐RELATED7* (*SMR7*), *NITRATE TRANSPORTER 2.1* (*NRT2.1*), and *SMR5*) in the *atx1setm* mutant. The RT‐qPCR and RNA‐seq data showed a similar transcript abundance trend for the evaluated genes (Fig. S7). The consistency of the results obtained by both methods validated our analysis of the identified DEGs.

Among the downregulated DEGs involved in root development, we identified, as putative targets of ATX1 (Fig. 4c), the genes encoding plant‐specific transcription factors, such as *TEOSINTE BRANCHED*/*CYCLOIDEA*/*PROLIFERATING CELL FACTOR 2* (*TCP2*) and *TCP24* from the TCP gene family, which are involved in several developmental processes (Li, 2015), and *AGAMOUS‐LIKE14* (*AGL14*), *AGL79*, *AGL71*, and *AGL96*. The latter genes belong to the *MCM/AG/DEFICIENS*/*SERUM RESPONSE FACTOR* (MADS‐box) gene family, the members of which are involved in both shoot and root development (Alvarez‐Buylla *et al*., 2000a,b; Tapia‐López *et al*., 2008; Garay‐Arroyo *et al*., 2013; Gao *et al*., 2018) or mainly in root development (e.g., *AGL14*; Garay‐Arroyo *et al*., 2013, and *AGL79*; Gao *et al*., 2018). Other identified DEGs upregulated in the *atx1setm* background were reported to be involved in LR development (Fig. 4c), such as *LBD29* (Porco *et al*., 2016), *LBD33* (Berckmans *et al*., 2011), and *NITRATE TRANSPORTER2.1* (*NRT2.1*; Little *et al*., 2005).

Overall, the transcriptomic analysis revealed potential targets of ATX1 that are essential for plant development.

### Putative ATX1 targets involved in LR development

A previous study (Napsucialy‐Mendivil *et al*., 2014) and this work strongly suggest that ATX1 is required for LR development, specifically for LRP morphogenesis, cell cycle timing control, and coordination of cell proliferation and cell growth. LR development in *A. thaliana* starts in the xylem‐pole pericycle (XPP) cells (Laskowski *et al*., 1995; Dubrovsky *et al*., 2000; Beeckman *et al*., 2001), whose transcriptional profile differs significantly from the phloem‐pole pericycle (PPP) (Parizot *et al*., 2012). As XPP marker (J0121) is also expressed at early LRP stages (Laplaze *et al*., 2005; Dubrovsky *et al*., 2006), DEGs identified by Parizot *et al*. (2012) as enriched in XPP also included DEGs enriched in LRP cells. Thus, with the aim of identifying possible ATX1‐dependent players involved in LRP formation, we searched for downregulated DEGs identified in *atx1setm* roots by RNA‐seq and compared them with published data (Parizot *et al*., 2012) on transcripts preferentially expressed in XPP or PPP (Fig. 4d). These DEGs have different functions, which explains the pleiotropic phenotype of the *atx1setm* mutant. For example, some DEGs were preferentially expressed in XPP cells (Parizot *et al*., 2012), such as those encoding phosphoglycerate mutase (*dPGM*), peroxidase (*PRX35*), transcription factor (*NAC103*), and carboxylase (*ACC2*), whereas others were preferentially expressed in PPP cells, such as those encoding E3 ubiquitin ligase (*PUB19*), Nodulin MtN21‐like transporter (*UMAMIT20*), and a hydrolase, AT5G06570 (GEO accession no.: GSE252067). These genes were also enriched in other cell types (Fig. S8), such as phloem/pericycle and a sub‐cell type of stele as categorized in Zhang *et al*. (2019).

One gene, *PRX35*, was particularly intriguing because (a) its expression level in *atx1setm* was significantly lower than that in the WT (Fig. 4d) and (b) *PRX35* could correspond to simultaneously more than one of the most enriched Gene Ontology categories that include cell wall organization, response to oxidative stress, hydrogen peroxide catabolic processes, and positive regulation of development (Fig. 4b). In addition, our preliminary experiments showed that a *PRX35* loss‐of‐function mutant had a peculiar LRP phenotype. Therefore, we studied *PRX35* as a putative ATX1 target.

### *PRX35* is a new player in LRP morphogenesis

**Fig. 5:**
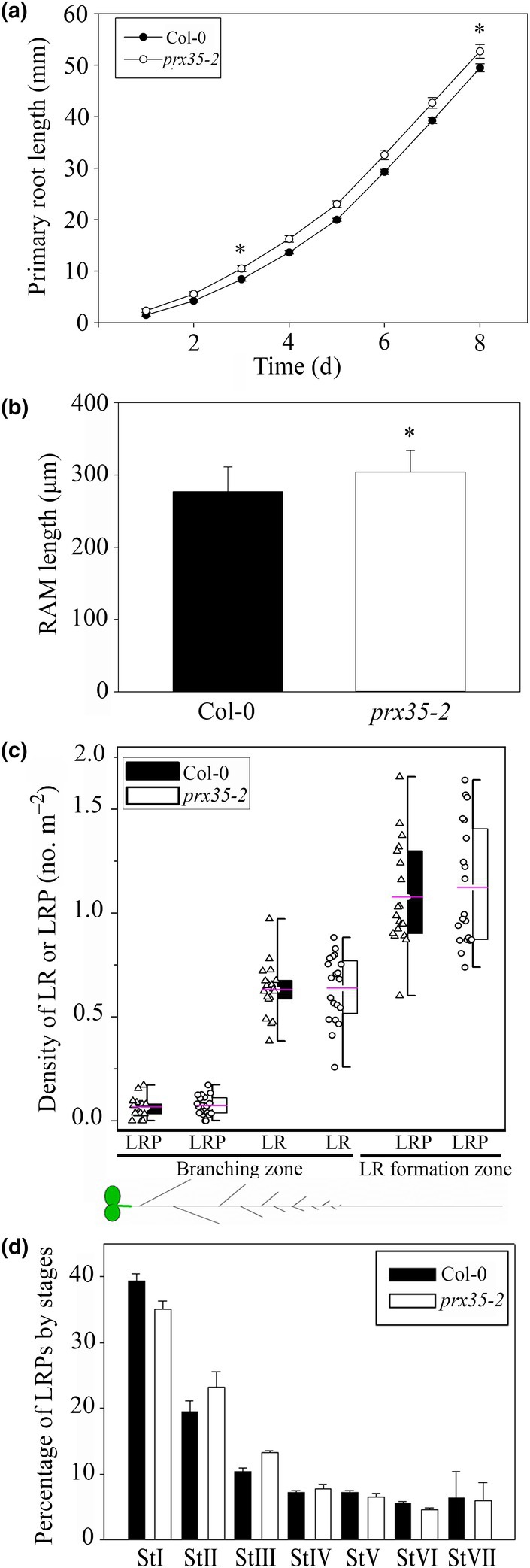
Phenotype of the *prx35‐2* mutant of *Arabidopsis thaliana*. (a) Primary root growth of wild‐type (Columbia‐0 (Col‐0)) and *prx35‐2* seedlings. Mean ± SE; * indicates statistically significant difference for root lengths at *P* = 0.020 and *P* = 0.049, Student's *t*‐test, three and 8 d after germination, respectively. (b) Root apical meristem (RAM) length in Col‐0 and *prx35‐2* seedlings. Two independent experiments. *n* = 21–22. Mean ± SD, * indicates a statistically significant difference at *P* = 0.008 (Student's *t*‐test). (c) Quantitative analysis of lateral root (LR) formation in Col‐0 and *prx35‐2* seedlings. In all cases, *P* > 0.05, Student's *t*‐test; all data and mean values are shown, boxes span from 25 to 75% percentiles, and whiskers range from a minimum to a maximum value. (d) Percentage of lateral root primordia (LRPs) by stage in Col‐0 and the *prx35‐2* mutant in the LR formation zone. Two independent experiments, *n* = 19–20. Mean ± SE. No statistically significant difference was observed (*P* > 0.05, Student's *t*‐test). All results are for seedlings at 8 d after germination.

*PRX35* (AT3G49960) encodes class III plant peroxidase, and we explored its role in LR formation as a case study of an ATX1‐regulated gene involved in root development. First, we determined the abundance of *PRX35* transcript in the roots of homozygous loss‐of‐function *prx35‐2* mutant plants (Fig. S9). No *PRX35* mRNA was detected in root tissues. Next, we studied the root phenotype of this mutant. The primary root length in 3–8 dag *prx35‐2* seedlings was slightly but significantly longer compared with the WT (Fig. 5a). In agreement with this, the RAM was longer in the *prx35‐2* mutant compared with the WT (Fig. 5b). To substantiate these findings, we analyzed the primary root development of a second allelic mutant, *prx35‐1* (Jeong *et al*., 2022) and found a similar phenotype (Fig. S10a,b). These data suggest that changes in ROS balance affect primary root growth in the *prx35* mutants.

As the *atx1setm* mutant was markedly affected in LR development and morphogenesis, we focused our analysis on similar processes in the *prx35‐2* mutant. No changes in the density of LRs and LRPs were found (Fig. 5c), and the length of fully elongated cortical cells in *prx35‐2* was the same as in the WT (Fig. S11), indicating that LRI was unaffected in this mutant, similarly to *atx1* mutants (Napsucialy‐Mendivil *et al*., 2014 and this study). Analysis of the distribution of LRPs by developmental stages showed no differences in *prx35‐2* plants compared with the WT (Fig. 5d), suggesting that primordium formation was not delayed in this mutant.

**Fig. 6:**
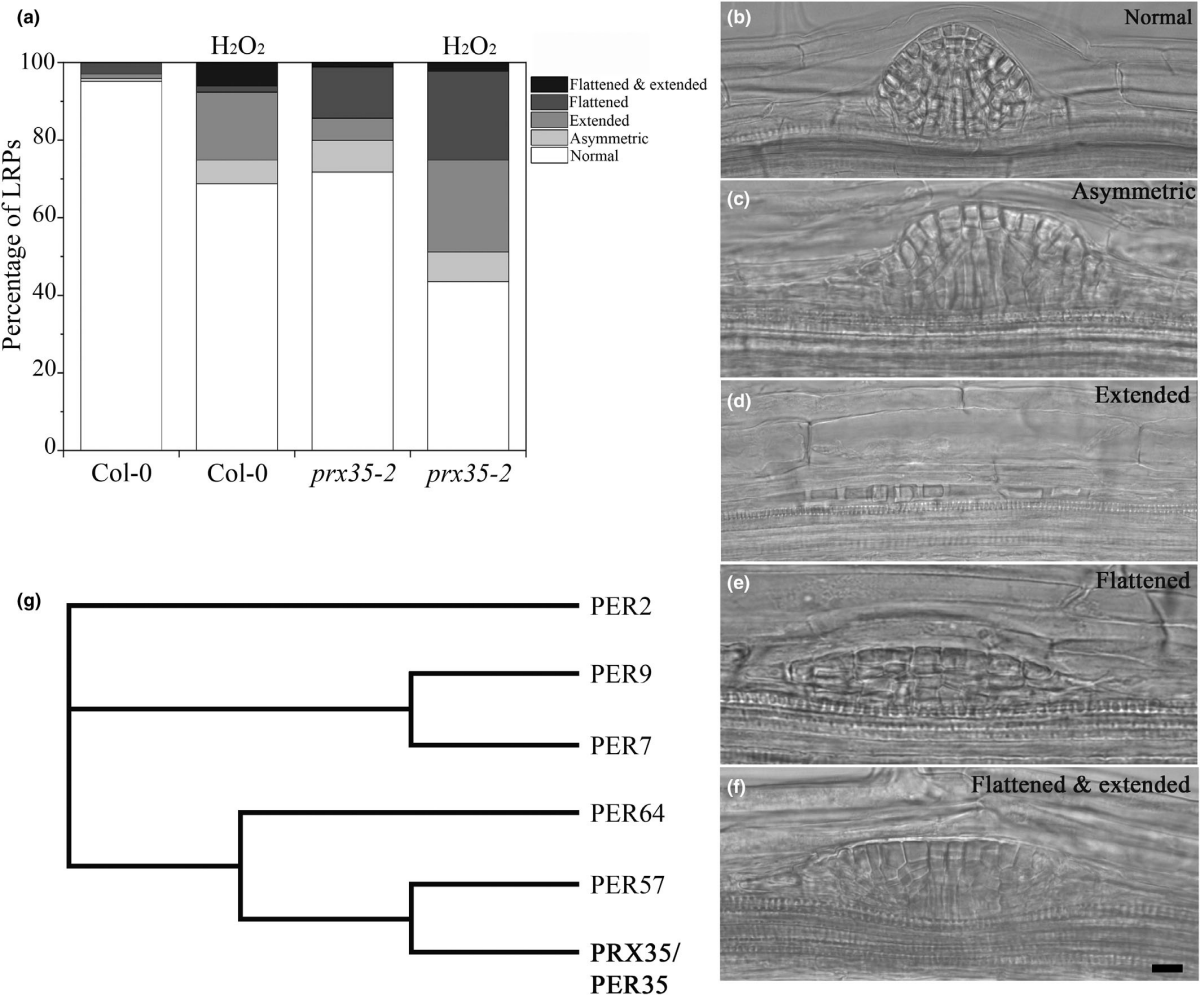
Lateral root (LR) primordium (LRP) morphogenesis in the *prx35‐2* mutant of *Arabidopsis thaliana*. (a) Percentage of abnormal LRPs in the LR formation zone wild‐type (Columbia‐0 (Col‐0)) and *prx35‐2* seedlings treated or not with 2 mM H_2_O_2_. Seedlings at 5 d after germination were transferred to a medium supplemented with 2 mM H_2_O_2_ where they grew for three additional days. Two independent experiments were performed; *n* = 19–22. (b) LRPs in the Col‐0. (c–e) Abnormalities in LRP formation in the *prx35‐2* mutant. All results are for seedlings at 8 d after germination. Bar, 20 μm (the same for (b–e)). (f) Cladogram of some members of the *A. thaliana* peroxidase protein family related to LR development.

We found that abnormal LRPs were more abundant in the *prx35‐2* mutant (29%) than in the WT (5%; Fig. 6a). In contrast to the LRP domes typical of WT roots (Fig. 6b), the *prx35‐2* LRP domes were often asymmetric or flattened (Fig. 6a). Additionally, we found the LRPs with longitudinally extended base and LRPs with an extended base and a flattened dome (Fig. 6a). Similar abnormalities were found in *prx35‐1* LRPs (Fig. S10c–f). These abnormal phenotypes morphologically resembled those found in the *atx1‐1* and *atx1setm* mutants (Napsucialy‐Mendivil *et al*., 2014, Fig. 3). The *prx35* mutants are expected to have a higher endogenous level of H_2_O_2_, causing these abnormalities. To establish whether this effect can be exacerbated by an exogenous application of H_2_O_2_, we transferred 5 dag *prx35‐2* and WT seedlings to 0.2× MS medium supplemented with 2 mM H_2_O_2_ and grew them for an additional 3 d. This concentration was selected because our preliminary experiments showed that no effect was detected with a lower concentration of H_2_O_2_. The analysis was carried out specifically in the LR formation zone, where LRPs are initiated and developed during the treatment. In the *prx35‐2* mutant treated with 2 mM H_2_O_2_, we found a 25% increase in abnormal LRPs compared with untreated seedlings (Fig. 6a). Noteworthily, the percentage of abnormal LRPs in WT seedlings treated with H_2_O_2_ was similar to that in untreated *prx35‐2* seedlings (Fig. 6a; *P* > 0.05; Student's *t*‐test), indicating that H_2_O_2_ treatment in the WT phenocopied the mutant abnormalities. Together, these results indicate that *PRX35* has an essential role in LRP morphogenesis.

Other peroxidases are known to be involved in LR development (Manzano *et al*., 2014; Orman‐Ligeza *et al*., 2016; Fernández‐Marcos *et al*., 2017). Therefore, the amino acid sequences of PER2, PER7, PER57, PER9, and PER64 proteins, which are all involved in LR development (Manzano *et al*., 2014; Fernández‐Marcos *et al*., 2017) (https://www.ebi.ac.uk/Tools/msa/clustalo/), were aligned with that of PRX35, and their levels of identity and similarity were analyzed (Fig. 6g). Strikingly, a low percentage of identity was found among these sequences. For example, PRX35 is more closely related to PER57 (42.3%) and PER64 (42.6%) than to the other members examined (Fig. 6g). This result suggests that there is low functional redundancy between these six peroxidases. Although there are 73 peroxidase genes in the *A. thaliana* genome (Cosio & Dunand, 2009), the phenotype of the *prx35* loss‐of‐function mutants, which have a flat‐dome‐shaped primordia and other abnormalities (Figs 6b–f, S10c–h), supports the notion that PRX35 activity is essential for normal LR morphogenesis. Also, we analyzed genes that are co‐expressed with *PRX35* (https://bar.utoronto.ca/) and found many genes involved in cell wall remodeling, such as *PECTIN METHYLESTERASE46* (*PME46*), which regulates pectin esterification between pericycle and endodermal cells; this enzymatic activity is essential for LRI (Wachsman *et al*., 2020; Table S3). Notably, *PME46* and the other seven genes involved in cell wall remodeling were co‐expressed with *PRX35* and were also downregulated in the *atx1setm* transcriptome data (Table S3, marked in bold). These results suggest that *PRX35* is part of a regulatory network that is important for LR development.

**Fig. 7:**
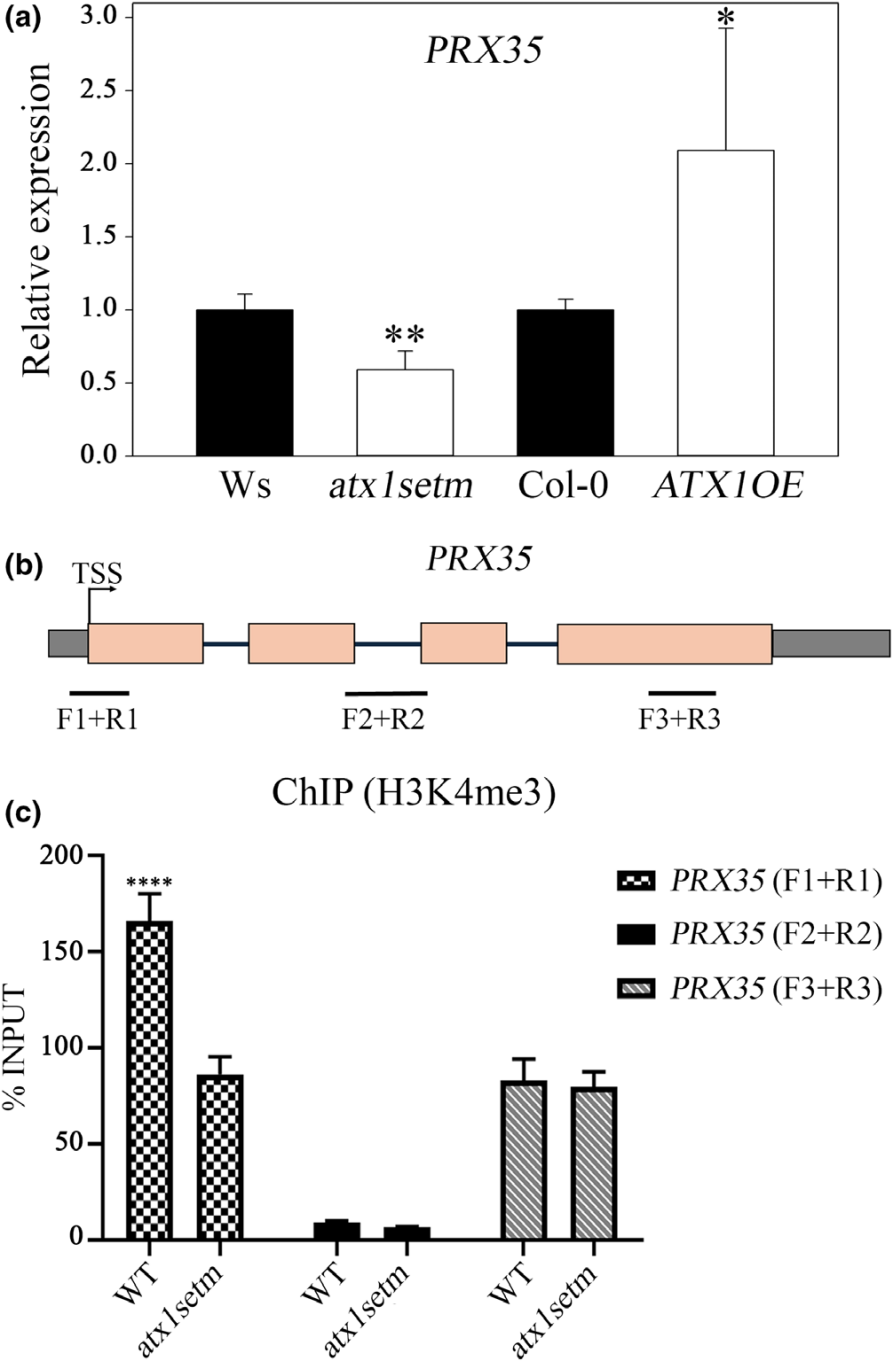
ARABIDOPSIS HOMOLOG OF TRITHORAX1 (ATX1) promotes *PRX35* expression in *Arabidopsis thaliana*. (a) Reverse transcription quantitative polymerase chain reaction (RT‐qPCR) quantification of transcript abundance in the wild type (WT) (Wassilewskija (Ws) and Columbia‐0 (Col‐0)), *atx1setm* mutant, and *35S::ATX1*overexpressing (ATX1OE) lines in roots at 8 d after germination. Mean data ± SD are from two biological replicates with three technical replicates. Significant difference was determined by Student's *t‐*test (**, *P* = 0.001; *, *P* = 0.002). (b) Schematic diagram of *PRX35* and PCR amplicons used for chromatin immunoprecipitation (ChIP)‐qPCR. TSS: transcription start site; amplicon positions are indicated as F + R (for forward and reverse primers of each amplicon). (c) Histone methylation profile. ChIP assays to determine the presence or absence of the histone H3 lysine‐4 trimethylation mark (H3K4me3) at three different regions within the *PRX35* locus. Data are means ± SD. Each ChIP experiment was performed for two biological replicates (each with two technical replicates). Statistical significance (****) was determined by a Brown–Forsythe test (*P*‐value < 0.0001).

To assess whether the *PRX35* expression could be promoted by ATX1 activity, we performed an RT‐qPCR analysis of *PRX35* transcript abundance in the *atx1setm* mutant and *35S::ATX1* overexpression line (*ATX1OE*; Alvarez‐Venegas *et al*., 2003). We found a decrease in the transcript level of *PRX35* in the *atx1setm* background, where the ATX1 activity was compromised. In accordance with this result, the transcript level of *PRX35* was increased in the *ATX1OE* background (Fig. 7a). As expected, the *PRX35* abundance in the *atx1‐1* mutant was significantly decreased (Fig. S12). These results support the transcriptomic data, suggesting that ATX1 promotes *PER35* expression.

Next, we investigated whether changes in the *PER35* expression were associated with changes in its chromatin structure. To this end, we performed ChIP assays in WT and *atx1setm* seedling roots using an anti‐H3K4me3 antibody. We examined three distinct regions of *PRX35*: the promoter–exon boundary region, Intron 2, and Exon 4 (also referred to as the 3′ −1 nucleosome; Fig. 7b). This analysis revealed a statistically significant enrichment of the H3K4me3 mark at the nucleosome putatively positioned at the promoter–exon boundary region in the WT, with a 165‐fold increase compared with an 80‐fold increase in *atx1setm* roots (normalized to the input sample of chromatin before immunoprecipitation; Fig. 7c). Despite this reduction in H3K4me3 enrichment at this region, *atx1setm* roots still exhibited the H3K4me3 mark at the +1‐nucleosome region when normalized to the input sample. By contrast, there was no enrichment for the H3K4me3 mark at the intronic region (amplified by the *PRX35* F2 + R2 primer set), indicating a lack of nucleosomes in this region, as expected (Fig. 7c). WT and *atx1setm* lines displayed a similar enrichment of the H3K4me3 mark at exon 4 (3′ −1 nucleosome; Fig. 7b), which was comparable to the enrichment at the +1‐nucleosome region in the *atx1setm* plants. As expected, the decrease in H3K4me3 mark enrichment at the promoter–exon boundary region in *atx1setm* roots was related to low *PRX35* expression levels. This finding indicates that the reduced deposition of H3K4me3 at the promoter–exon boundary of *PRX35* is linked to the downregulation of the *PRX35* expression in *atx1setm* mutant.

Together, our data suggest that ATX1 impacts LR development through epigenetic regulation of its targets, some of which are involved in LRP morphogenesis and root system architecture.

## Discussion

The role of epigenetic regulation in plant developmental processes is well established (Feng *et al*., 2010; He *et al*., 2011; Pikaard & Scheid, 2014; Kumar & Mohapatra, 2021), but the developmental control of LR development is yet to be determined. Importantly, in mutants compromised in ATX1, LRI is maintained the same as in the WT, and only LRP morphogenesis is affected. We previously showed that ATX1 is essential for LRP morphogenesis (Napsucialy‐Mendivil *et al*., 2014). Here, we attempted to discern possible mechanisms underlying the role of ATX1 in this process. Our data show that when the activity of the ATX1 catalytic SET domain is abolished, the extent of abnormalities in LRP formation is greater compared with a mutant devoid of the ATX1 protein, underlying the importance of methyltransferase activity in LRP morphogenesis. We also explored which particular LRP morphogenesis processes are affected in *atx1* mutants. This analysis showed that ATX1 is required for normal LRP morphogenesis through different mechanisms. Even though LRP initiation was not affected in *atx1* mutants at a quantitative level, it was qualitatively compromised. The *atx1* mutants can form abnormal multilayered early‐stage LRPs without a pronounced dome (Fig. 3a, third panel), suggesting that the differences in proliferation potential between the core and flanks of an LRP (Hernández‐Herrera *et al*., 2022; Stöckle *et al*., 2022) are not maintained. The extended LRP base in these mutants suggests that longitudinally a greater number of founder cells participate in the LRP initiation (Fig. S3b) and that the morphogenetic field boundaries are not perceived properly (Torres‐Martínez *et al*., 2020). Lateral inhibition mechanisms prevent closely spaced LRP initiation and maintain a certain average distance between the initiation events taking place in an acropetal pattern (Dubrovsky *et al*., 2006; Toyokura *et al*., 2019). Closely spaced LRP initiation in the mutants (Fig. S3e) suggests that these mechanisms are compromised in these plants. In addition to qualitatively affected LRI, LRP morphogenesis becomes severely affected and delayed because of (a) an extended cell cycle time, (b) asymmetric dome formation, (c) changed PGD, and (d) fused LRs (Fig. 3). These abnormalities also explain why in a fraction of *atx1setm* mutant seedlings LRs did not emerge. Our data showed that CDK inhibitor genes *SMR5* and *SMR7* were some of the most upregulated DEGs in *atx1‐1setm* (Figs S6a, S7). Higher expression of these genes results in an extended cell cycle duration (Yi *et al*., 2014); therefore, their upregulation in *atx1‐1setm* can explain the longer cell cycle during LRP formation in the *atx1* mutants. Another upregulated cell cycle related gen was *TSO2* (AT3G27060) that encodes a ribonucleotide reductase involved in control of cell proliferation and DNA damage (Wang & Liu, 2006).

To further explore the possible mechanisms underlying the abnormal development in the *atx1* mutants, we performed a transcriptomic analysis (Fig. 4) of the root tissues of the *atx1setm* mutant. Among the enriched Gene Ontology categories for genes downregulated in *atx1setm* were plant‐type cell wall organization and hydrogen peroxide catabolic process. Cell wall modifications and remodeling are closely linked to LR morphogenesis and emergence (Kumpf *et al*., 2013; Wachsman *et al*., 2020; Winter *et al*., 2023). The transcriptomic approach led us to identify genes implicated in LR development that are regulated by ATX1 at the epigenetic level (Fig. 4d). Some of the downregulated genes could be direct targets of ATX1 activity. Among them, we found transcription factors, transporters, carboxylases, hydrolases, and peroxidases, all of which can potentially be involved in LR development. The diverse functions of the affected genes can explain the pleiotropic phenotype of the *atx1setm* mutant (Figs 1, 3).

As the focus of this study was on LRP morphogenesis, we searched for candidate DEGs that could be required for this developmental process. Preliminary analysis of loss‐of‐function mutants in some of these genes suggested that *PRX35* might be regulated by ATX1. The abnormal LRP morphogenesis identified in the *prx35* mutants (Figs 5, 6, S10) was similar to that in the *atx1‐1* and *atx1setm* mutants, which showed a decreased *PRX35* abundance (Figs 7a, S12). These results suggested that *PRX35* transcription could indeed be controlled by ATX1 due to its methyltransferase activity (Fig. 7c). The finding that the percentage of abnormal LRPs in the *prx35* mutants is lower than in *atx1setm* (compare Fig. 3b with Fig. 6a) suggests that other genes whose active transcription depends on ATX1 can also be involved in LRP morphogenesis control. These data provide a link between LRP morphogenesis, ATX1 activity, and the role of PRX35 during root formation.

Members of the chromatin remodeling complex, such as SWR1 belonging to TrxG, are involved in the maintenance of RAM homeostasis, and oxidoreductase activity‐related gene expression is significantly downregulated in mutants of the SWR1 complex members. These changes were accompanied by decreased H3K4me3 enrichment and increased H3K27me3 enrichment in the respective mutants (Huang *et al*., 2023). In general, ROS signaling and redox state are central to many processes in plant development (Gilroy *et al*., 2016; Mase & Tsukagoshi, 2021; Peláez‐Vico *et al*., 2024), and peroxidases have a vital role during LR development (Manzano *et al*., 2014; Orman‐Ligeza *et al*., 2016).

Various ROS are essential for LR formation. Plant NADPH oxidases called Respiratory Burst Oxidase Homologs (RBOHs) generate superoxide anion followed by its dismutation to oxygen peroxide (Smirnoff & Arnaud, 2019). In *Phaseolus vulgaris*, PvRbohB and PvRbohA were shown to be involved in the maintenance of LR density (Montiel *et al*., 2012) and LR emergence (Arthikala & Quinto, 2018). Similarly, in *A. thaliana*, different RBOH genes are required for LR emergence, and this effect is mediated by cell wall remodeling in the endodermal, cortical, and epidermal cells overlying the developing LRP (Orman‐Ligeza *et al*., 2016). Furthermore, H_2_O_2_ promotes LR emergence (Manzano *et al*., 2014; Orman‐Ligeza *et al*., 2016), while peroxidases participate in H_2_O_2_ elimination. Here, we show that in the *prx35* mutants, neither primary root growth (Figs 5a, S10a) nor LR density (Fig. 5c) were negatively affected compared with the WT, suggesting that the level of endogenous increase in H_2_O_2_ in this mutant was not significantly elevated in the root of these mutants. However, the *prx35* LRP morphogenesis was abnormal (Figs 6a–e, S10c–h), which could be explained by the H_2_O_2_ involvement in cell wall remodeling, influencing cell wall loosening and in pectin biosynthesis and modifications (O'Brien *et al*., 2012; Gall *et al*., 2015; Xiong *et al*., 2015; Barnes & Anderson, 2018; Mase & Tsukagoshi, 2021; Zhang *et al*., 2022). Importantly, *PRX35* was co‐expressed with many genes related to cell wall remodeling (Table S3). Also, H_2_O_2_ is involved in a redox homeostasis required for the quiescent center establishment and function (Kerk & Feldman, 1995; Jiang *et al*., 2003), which could be altered in the mutant, thereby affecting LRP morphogenesis and new RAM establishment. Interestingly, despite that in *c*. half of all 8 dag *atx1setm* plants LRs did not emerge (Fig. 2), other peroxidases involved in LR emergence (Fig. 6g) were not found among *atx1setm* DEGs, suggesting that ATX1 does not maintain their active transcription. Our results show that changes in *PRX35* expression were associated with changes in its chromatin structure. Specifically, the H3K4me3 levels in the *PRX35* promoter region were lower in the *atx1setm* roots than in the corresponding WT. This indicates that ATX1 is involved in active *PRX35* transcription. Furthermore, the enrichment of the H3K4me3 mark in the promoter region of *PRX35* was 50% lower in *atx1setm* plants than in the corresponding WT, suggesting that other SDG proteins also mediate H3K4me3 enrichment at this site (Fig. 7c). In *A. thaliana*, COMPASS‐like complexes associate with multiple H3K4 methyltransferases that could deposit H3K4me3 marks in the *atx1setm* mutant to promote expression of the target genes (Miller *et al*., 2001; Jiang *et al*., 2011; Shang *et al*., 2021). Stronger phenotype of the *atx1setm* that of *atx1‐1* mutant suggests that ATX1 with abolished methyltransferase activity competes for the common target loci. Decreased level of H3K4me3 methylation marks at the promoter region of *PRX35* and its reduced expression in *atx1setm* roots suggest their profound effect on LRP morphogenesis described in this work. Furthermore, the nucleosome occupancy seen at the last *PRX35* exon (3′ −1 nucleosome) could impact gene transcription levels or play a role in transcriptional plasticity (Chen *et al*., 2017). At this exon, the enrichment of H3K4me3 marks in the *atx1setm* mutant and the WT were similar, suggesting that the identified H3K4me3 marks result from the activities of other methyltransferases and that ATX1 is essential for the promotion of *PRX35* transcription.

In summary, we showed that ATX1 is involved in the epigenetic regulation of LRI and morphogenesis. Through transcriptomic analysis, we proposed candidate targets of ATX1. A link established in this study between *ATX1* and *PRX35* involved in redox homeostasis, revealed a novel aspect of epigenetic regulation of LR morphogenesis that operates both in the pericycle and its derivatives. How this link is related to cell cycle control and cell division orientation and the details of this link at the molecular level await further clarification.

## Competing interests

None declared.

## Author contributions

SN‐M and JGD designed the study. SN‐M performed most of the experiments. SN‐M, GR‐A and SS performed the transcriptome analyses. SN‐M and MAJ‐V generated the transgenic plants. DMR‐T and RA‐V performed the ChIP‐qPCR analysis. HHT‐M and SN‐M performed the time‐lapse experiments. SN‐M and JGD analyzed the data and wrote the manuscript. SS and JGD supervised the study.

## Disclaimer

The New Phytologist Foundation remains neutral with regard to jurisdictional claims in maps and in any institutional affiliations.

## Supporting information


**Fig. S1** Gene structure of *ATX1* and schematic representation of ATX1 protein domains.
**Fig. S2** Length of fully elongated cells and lateral root initiation index in wild‐type (Ws), *atx1‐1*, and *atx1setm* seedlings.
**Fig. S3** Abnormalities in lateral root development and lateral root primordium morphogenesis in the *atx1setm* mutant.
**Fig. S4** Percentages of abnormalities in lateral root primordium development in ATX1OE and Col‐0.
**Fig. S5** Developmental stages of lateral root primordia in Ws *TCTP1::3VENUS and atx1‐1 TCTP1::3VENUS* lines.
**Fig. S6** Venn diagrams of auxin‐ and cell‐cycle‐related genes in the *atx1setm* mutant.
**Fig. S7** Validation of selected differentially expressed genes.
**Fig. S8** Expression of selected genes of interest in cell populations of the wild‐type *Arabidopsis thaliana* root apex.
**Fig. S9** Genotyping of two mutants in *PRX35* gene and *PRX35* transcript abundance.
**Fig. S10** Phenotype of *prx35‐1* mutant.
**Fig. S11** Length of fully elongated cortical cells in Col‐0 and *prx35‐2* primary root.
**Fig. S12** Abundance of *PRX35* mRNA in the *atx1‐1* mutant background.
**Table S1** Primers used in this study.
**Table S2** Differentially expressed genes in *atx1setm* vs Ws.
**Table S3** Genes co‐expressed with *PRX35*.

**Video S1** Cell lineages of the lateral root primordia (LRPs) and their 3D visualization in a time‐lapse experiment.
Please note: Wiley is not responsible for the content or functionality of any Supporting Information supplied by the authors. Any queries (other than missing material) should be directed to the *New Phytologist* Central Office.

## Data Availability

Transcriptomic data were deposited in the Gene Expression Omnibus (GEO) of the National Center for Biotechnology Information and are accessible through GEO accession number GSE252067. The data that support the findings of this study are also available in the Supporting Information of this article.
