# Supplementary material for: ARABIDOPSIS HOMOLOG OF TRITHORAX1 impacts lateral root development by epigenetic regulation of targets involved in root system architecture
Source: New Phytol. 2025 Jul 7;247(5):2180–95. doi: 10.1111/nph.70349 (PMC12329177; doi:10.1111/nph.70349)
Supplement: Supplementary file 1 — Fig. S1 Gene structure of ATX1 and schematic representation of ATX1 protein domains. Fig. S2 Length of fully elongated cells and lateral root initiation index in wild‐type (Ws), atx1‐1, and atx1setm seedlings. Fig. S3 Abnormalities in lateral root development and lateral root primordium morphogenesis in the atx1setm mutant. Fig. S4 Percentages of abnormalities in lateral root primordium development in ATX1OE and Col‐0. Fig. S5 Developmental stages of lateral root primordia in Ws TCTP1::3VENUS and atx1‐1 TCTP1::3VENUS lines. Fig. S6 Venn diagrams of auxin‐ and cell‐cycle‐related genes in the atx1setm mutant. Fig. S7 Validation of selected differentially expressed genes. Fig. S8 Expression of selected genes of interest in cell populations of the wild‐type Arabidopsis thaliana root apex. Fig. S9 Genotyping of two mutants in PRX35 gene and PRX35 transcript abundance. Fig. S10 Phenotype of prx35‐1 mutant. Fig. S11 Length of fully elongated cortical cells in Col‐0 and prx35‐2 primary root. Fig. S12 Abundance of PRX35 mRNA in the atx1‐1 mutant background. Table S1 Primers used in this study. Table S2 Differentially expressed genes in atx1setm vs Ws. Table S3 Genes co‐expressed with PRX35. [file NPH-247-2180-s002.pdf]

New Phytologist **Supporting Information**

Article title: **ARABIDOPSIS HOMOLOG OF TRITHORAX1 impacts lateral root development by epigenetic regulation of targets involved in root system architecture**

Authors: Selene Napsucialy-Mendivil, Héctor H. Torres-Martínez, Gustavo Rodríguez-Alonso, Diana Marcela Rivera-Toro, Raúl Alvarez-Venegas, Marco Adán Júarez-Verdayes, Svetlana Shishkova, and Joseph G. Dubrovsky

Article acceptance date: 10 June 2025

The following Supporting Information is available for this article:

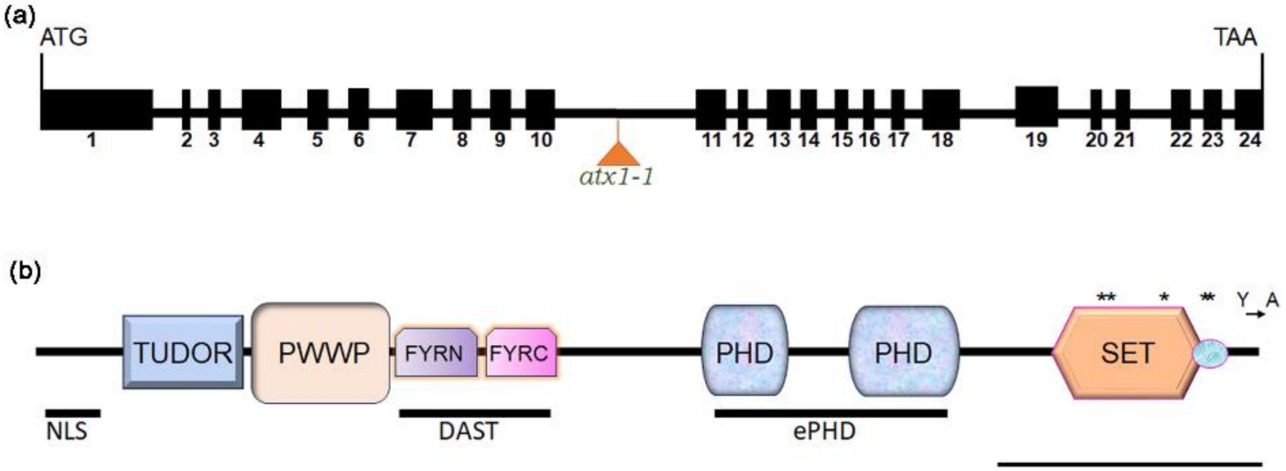

Fig. S1 Gene structure of *ATX1* and schematic representation of ATX1 protein domains
(a) Gene structure of *ATX1*. Numbered boxes indicate exons, and lines indicate introns. The triangle represents the T-DNA insertion site in the *atx1-1* mutant. (b) Schematic representation of ATX1 protein domains. The asterisks indicate the positions of the five amino acid substitutions [Tyr (Y) by Ala (A)] that inactivate the catalytic SET domain in the *atx1setm* mutant.

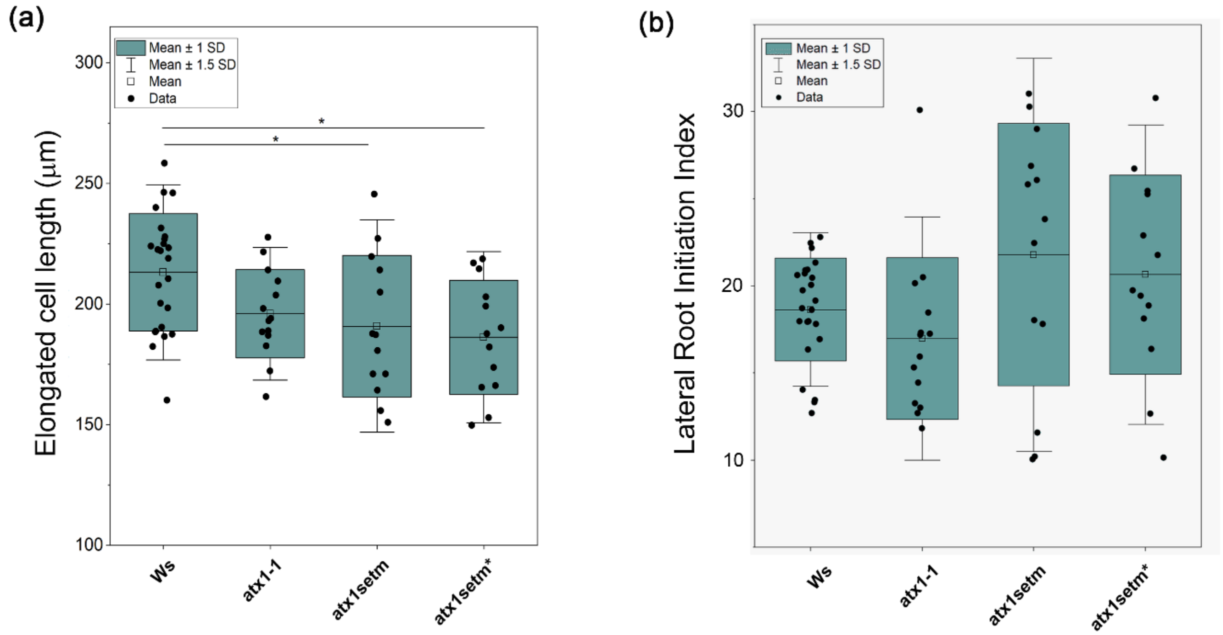

Fig. S2 Length of fully elongated cells and lateral root initiation index in wild-type (Ws), *atx1-1*, and *atx1setm* seedlings Subsample labeled *atx1setm** indicates plants in which no lateral roots (LRs) were present. (a) Length of fully elongated cortical cells. Asterisks indicate statistical difference at P<0.05; One-way ANOVA. (b) LR initiation index shows the number of all LR initiation events (LR and LR primordia) in a root portion corresponding to 100 cortical cells. One-way ANOVA showed no differences between samples (P = 0.073). For both plots, combined data of three independent experiments are shown; n ranges from 13 to 24 seedlings at eight days after germination; dots indicate individual data points; each data point in (a) shows average cell length of 10 cells.

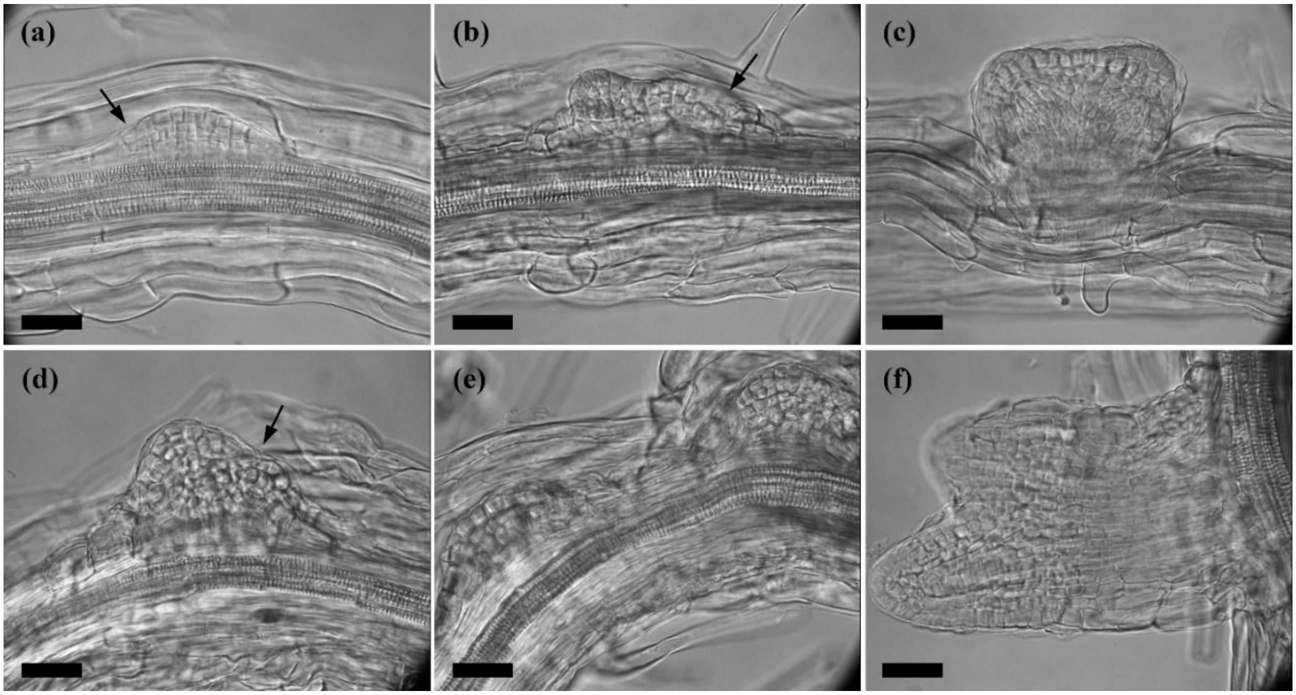

Fig. S3 Abnormalities in lateral root development and lateral root primordium morphogenesis in the *atx1setm* mutant Seedling roots were cleared at eight days after germination. Arrows denote the asymmetry in the lateral root primordium (LRP) dome (a, b, d). Asymmetric LRPs sometimes developed two domes that were fused as in (c) that eventually emerged as fused (fasciated) lateral roots as in (f). When two developing LRPs were not close enough to fuse, a short distance between them was maintained (e). Scale bars, 40 µm.

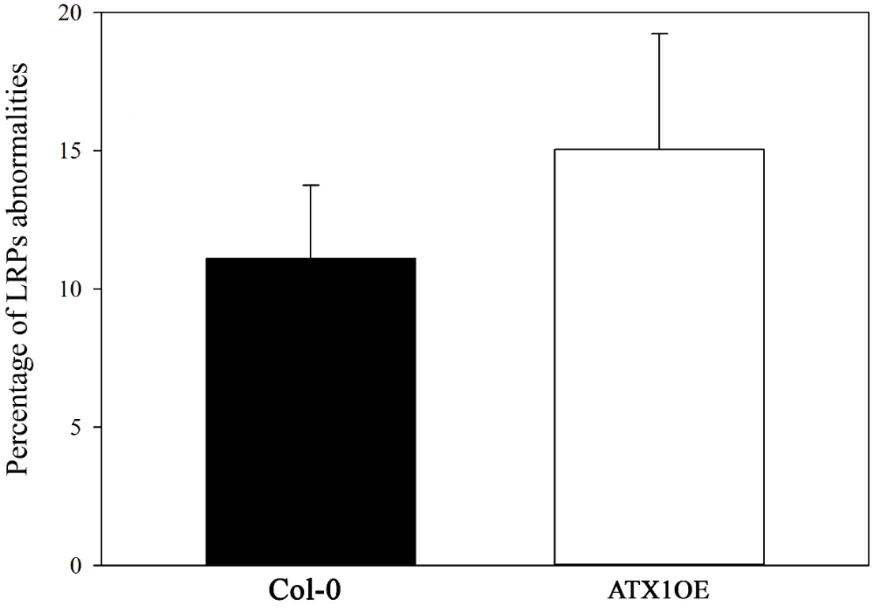

Fig S4. Percentages of abnormalities in lateral root primordium development in ATX1OE and Col-0 Mean ± SD; n = 7-11; eight days after germination. ATX1OE line was created by *atx1-1* complementation with *35S::ATX1*. No statistically significant difference was found, P = 0.477, Student's t-test.

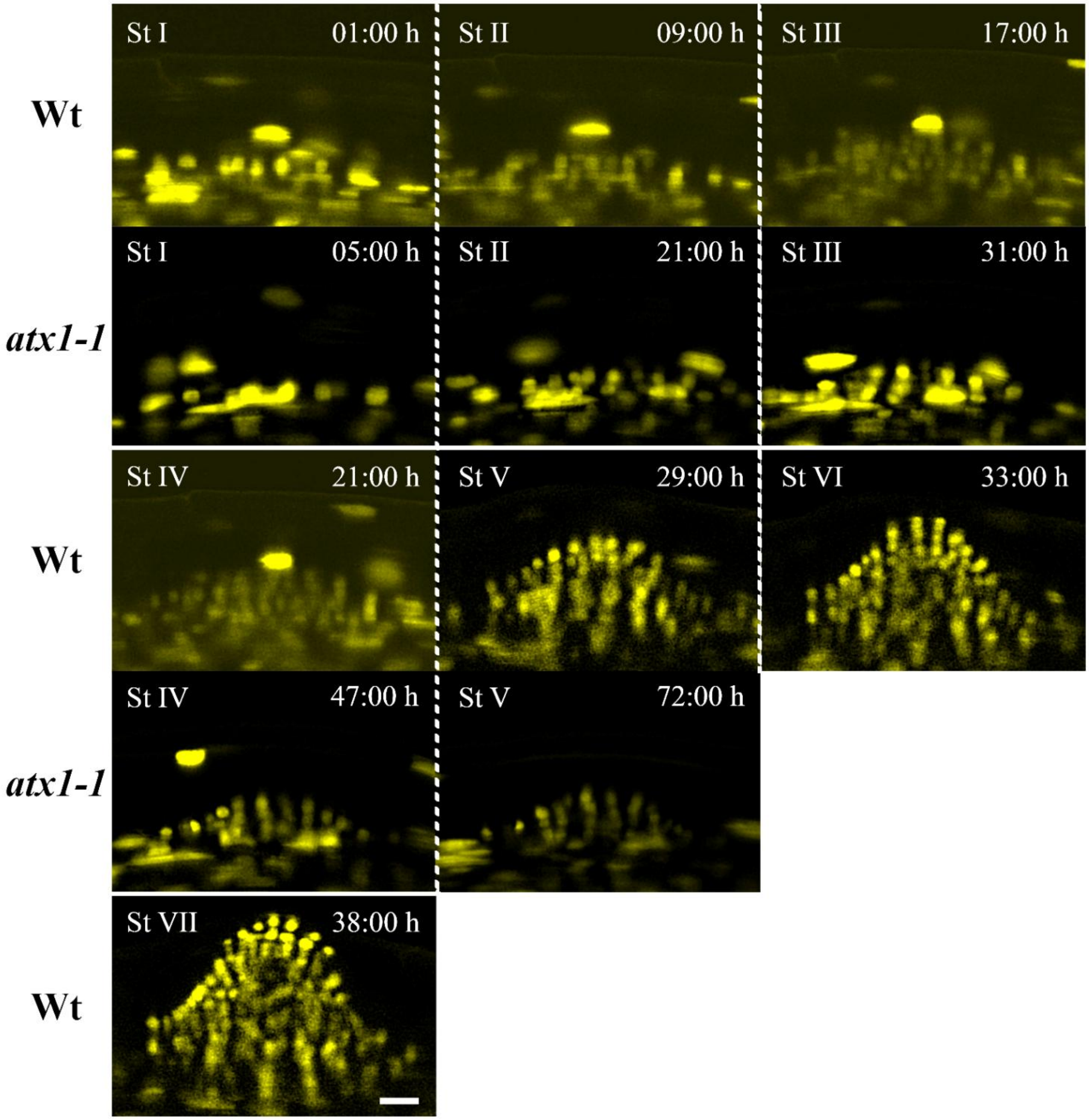

Fig. S5 Developmental stages of lateral root primordia in Ws *TCTP1::3VENUS and atx1-1 TCTP1::3VENUS* lines Representative images from one time-lapse experiment. In each panel elapsed time from the beginning of the experiment is shown; Stage I lateral root primordium of seedlings at five days after germination was selected as starting point. Each image represents a projection of 9 to 11 Z slices (4.5-5.5 µm total thickness). All panels were adjusted with increased exposure (+2) and gamma correction (1.1). Scale bar, 20 µm.

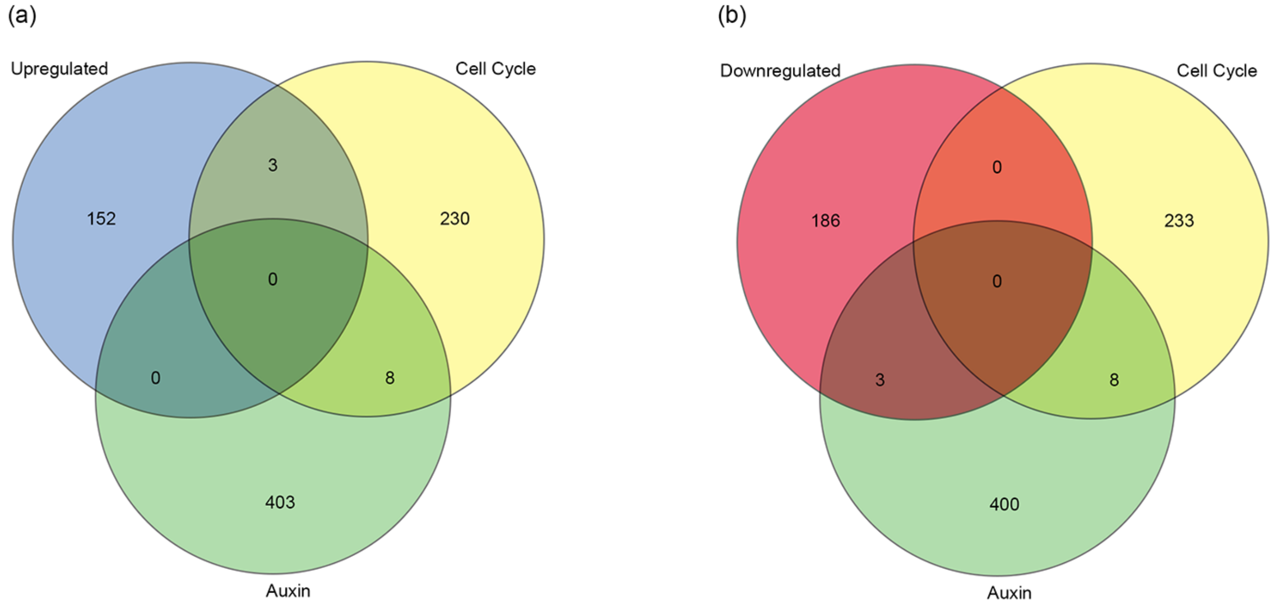

Fig S6. Venn diagrams of auxin- and cell-cycle-related genes in the *atx1setm* mutant The genes annotated as involved to auxin metabolism, perception or signaling and those cell-cycle-related were retrieved based on their gene ontology annotations. Venn diagram showing the number of up- and down-regulated genes in the *Arabidopsis atx1setm* vs wild type (Ws) transcriptome. Among up-regulated differentially expressed genes (DEGs) (a) and down-regulated DEGs (b) no genes involved in auxin metabolism, perception or signaling were found. No significant overlap between the up- and down-regulated genes in *atx1setm* and auxin- or cell-cycle-related genes was found. (a) The three genes in the overlap between the up-regulated and cell-cycle-related genes were *SIAMESE-RELATED5* (*SMR5,* AT1G07500), *SMR7* (AT3G27630, see Fig. S7) and *TSO2* (AT3G27060, see main text). (b) The three genes in the overlap between the down-regulated and indirectly related to auxin genes were *PROLINE-RICH PROTEIN* (AT3G62680), *XAANTAL2* (AT4G11880), and *ROOT HAIR DEFECTIVE 6-LIKE 4* (AT1G27740).

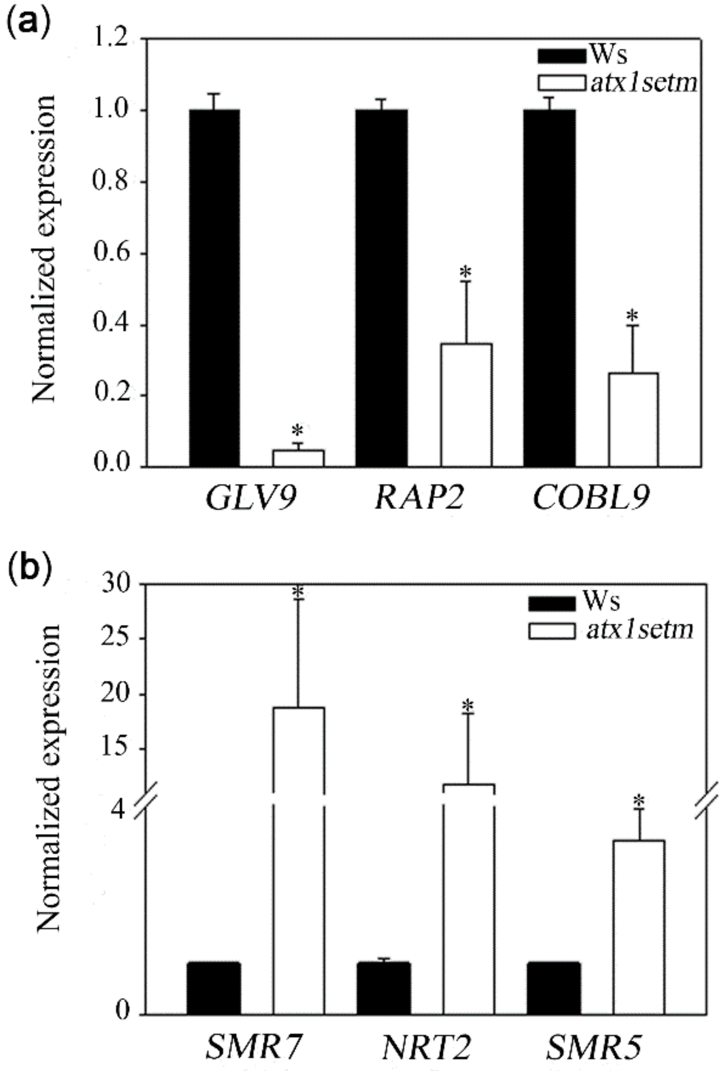

Fig. S7. Validation of selected differentially expressed genes Selected differentially expressed genes identified in the transcriptomic analysis of the *atx1setm* mutant versus the wild type (Ws) were validated with RT-qPCR. Three biological replicates with two technical replicates were performed using whole roots of seedlings at eight days after germination. Mean ± SD; * indicates a statistically significant difference at $P < 0.05$, Student's *t*-test. *UBQ10* and *EF1a* were used as reference genes. Selected genes: *GOLVEN9* (*GLV9*), *RELATED TO AP2.1* (*RAP2.1*), *COBRA-LIKE9* (*COBL9*), *SIAMESE-RELATED7* (*SMR7*), *NITRATE TRANSPORTER2* (*NRT2.1*), and *SMR5*.

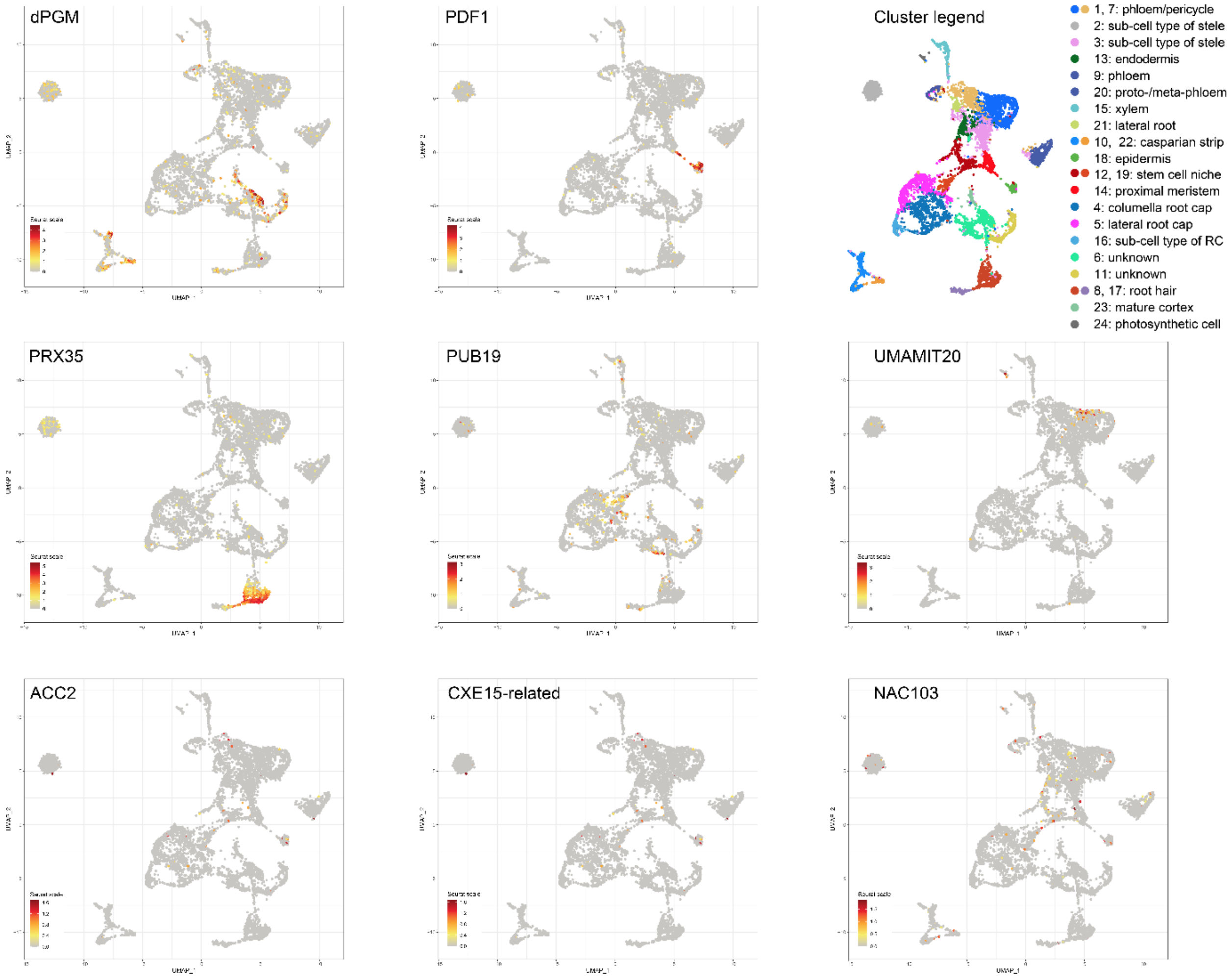

Fig. S8. Expression of selected genes of interest in cell populations of the wild type *Arabidopsis thaliana* root apex The selected genes were differentially expressed in the *atx1setm* mutant, which were previously reported as being preferentially expressed in the pericycle and some other cell types, such as phloem/pericycle and sub-cell type of stele (Parizot et al, 2012). Each dot represents a cell, and each cluster a population as shown in the figure legend (top right). The cluster identity is based on reference marker genes. Original data and categorization are from Zhang et al. (2019).

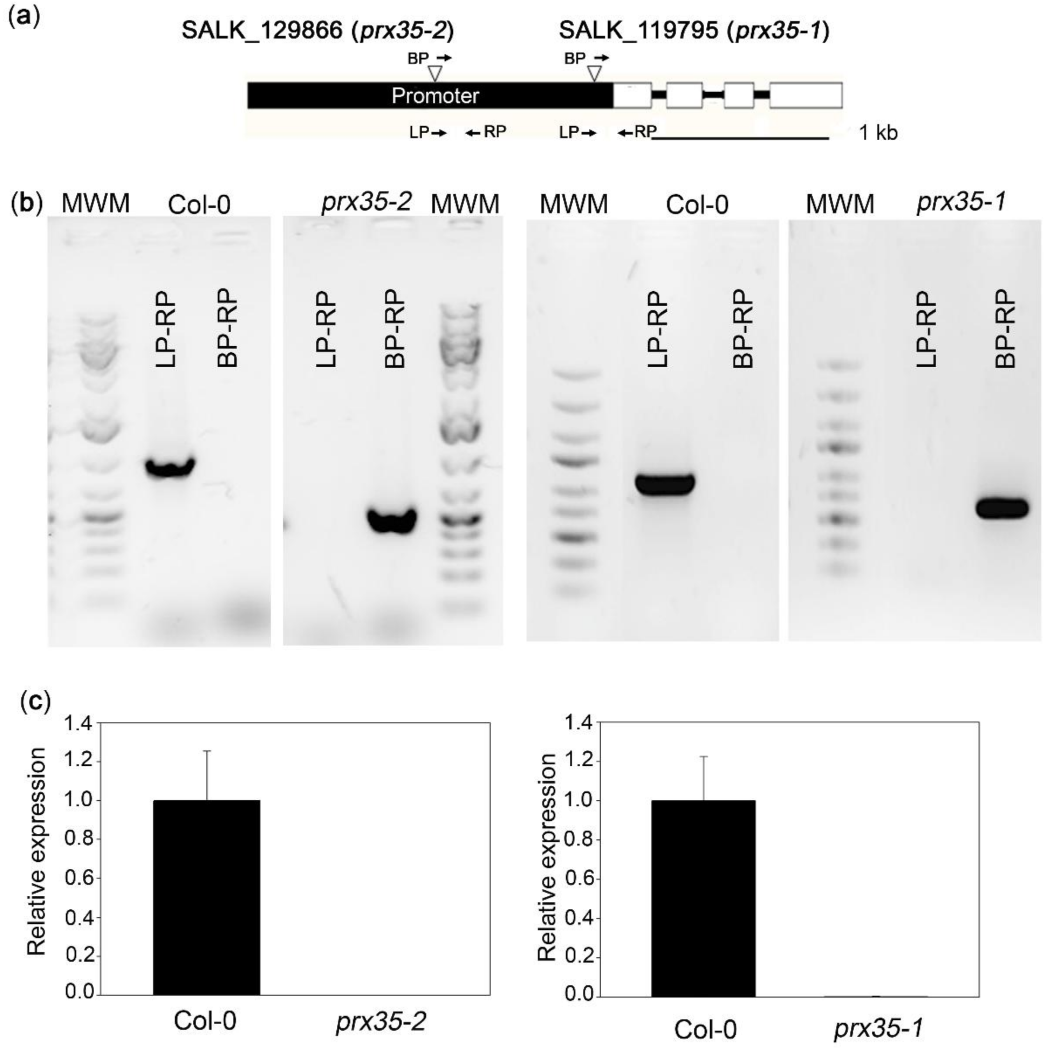

Fig. S9. Genotyping of two mutants in *PRX35* gene and *PRX35* transcript abundance (a) Gene structure of *PRX35*. The black box indicates the promoter, white boxes indicate exons, and lines indicate introns. The triangles in the promoter region indicate the location of T-DNA insertions in the two allelic mutations. (b) Genotyping of two *prx35* mutants. Genomic DNA was isolated, and PCR analyses were performed using gene-specific primers. The genotype was analyzed using the genomic primers (indicated for each allelic mutant as LP and RP) and a T-DNA primer LBb1.3 (indicated as BP); MWM, molecular weight marker. (c) Abundance of *PRX35* transcript in the *prx35* mutants, as determined by RT-qPCR. Two experiments were performed, three technical replicates each. Values are means ± SD; for both alleles $P<0.001$, Student's *t*-test.

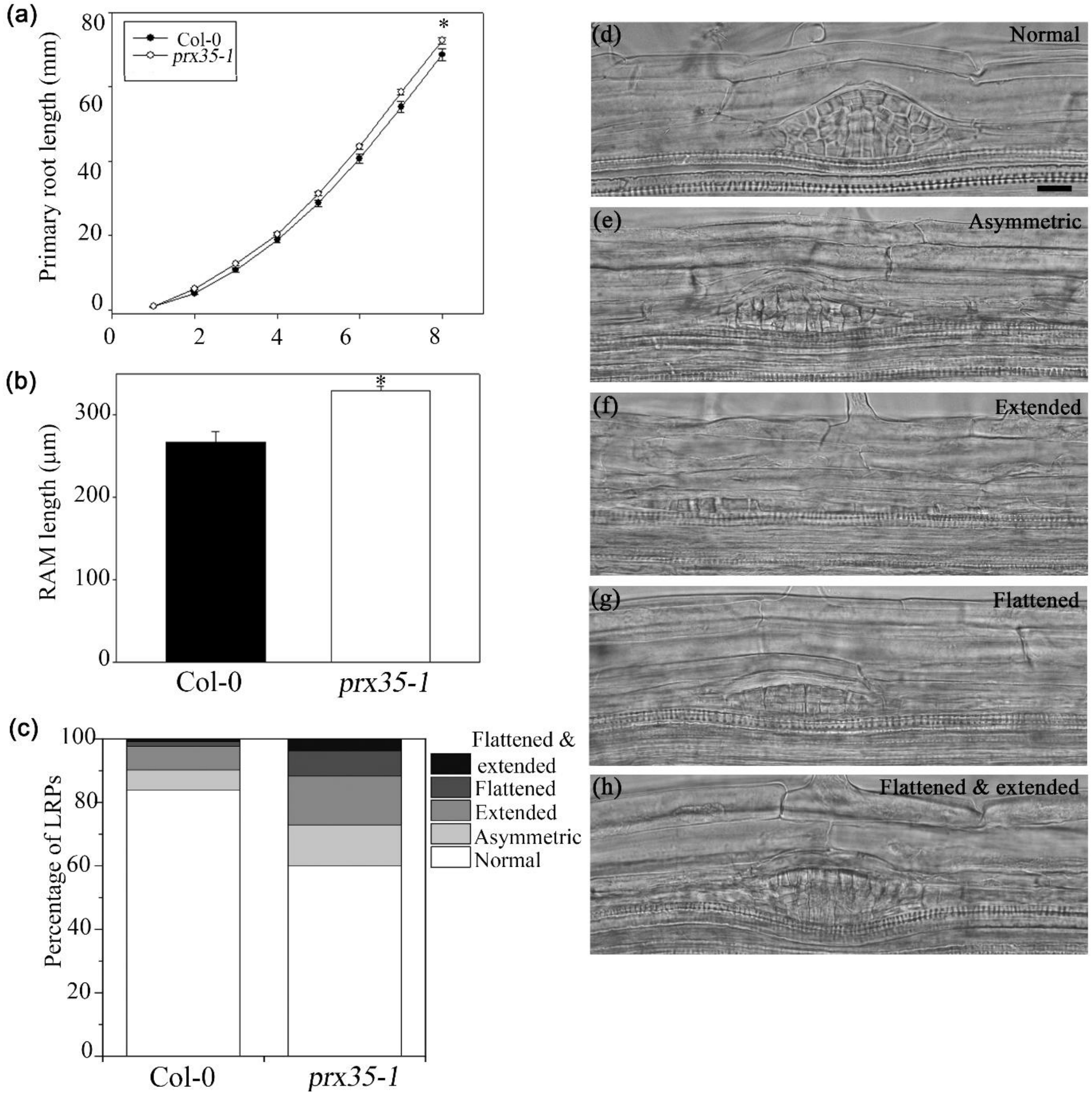

Fig. S10. Phenotype of *prx35-1* mutant. (a) Primary root growth of wild type (Col-0) and *prx35-1* seedlings. Mean± SE; * indicates statistically significant difference for root lengths at $P = 0.035$ (Student's *t*-test). (b) Root apical meristem (RAM) length in Col-0 and *prx35-1* seedlings. Col-0, $n = 8$; *prx35-1,* $n = 15$. Mean ± SE; * indicates a statistically significant difference at $P = <0.001$ (Student's *t*-test). (c) Percentage of normal and abnormal LRPs in Col-0 and the *prx35-1* mutant in the LR formation zone; Col-0, $n = 8$; *prx35-1,* $n = 15$. (d) A representative image of a normal lateral root primordium (LRP) in the Col-0. (e) to (h) images of categorized LRP abnormalities in the *prx35-1* mutant. All results are for seedlings at eight days after germination. Scale bar, 20 μm.

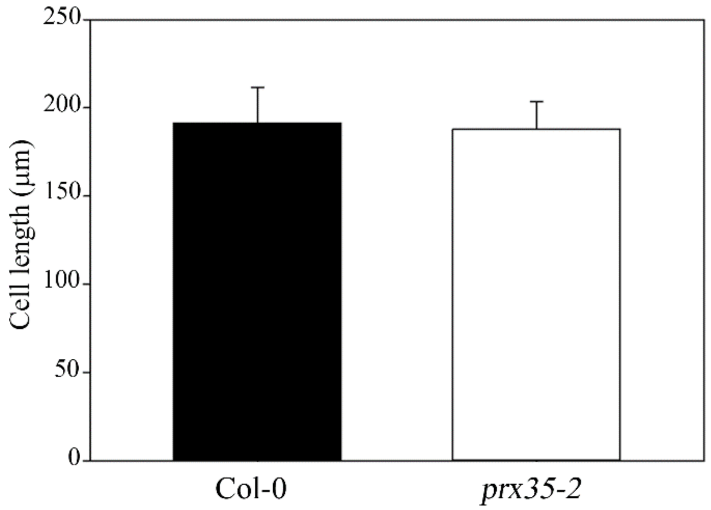

Fig. S11 Length of fully elongated cortical cells in Col-0 and *prx35-2* primary root Cell length was determined in the differentiation zone of seedlings at eight days after germination on cleared preparations. Two independent experiments; total n = 19–20 roots. Mean ± SD. No statistical difference was found ($P > 0.05$, Student's t-test).

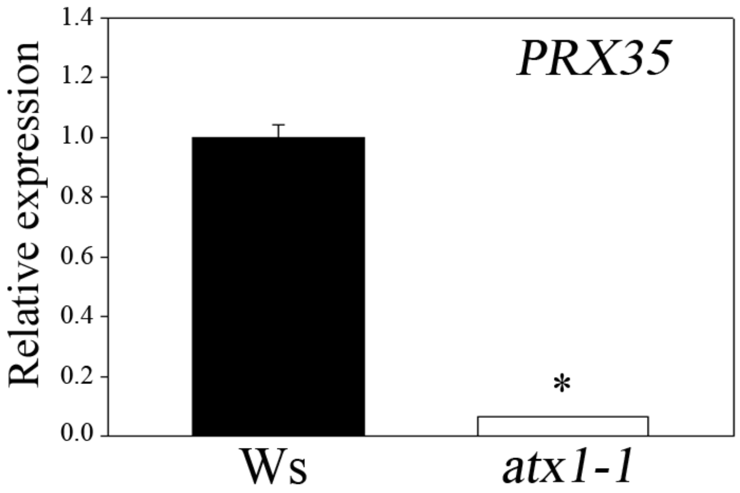

Fig. S12 Abundance of *PRX35* mRNA in the *atx1-1* mutant background The RT-qPCR was performed in one experiment with three technical replicates. Values are means ± SD. $P = <0.001$, Student's t-test.

Table S1. Primers used in this study

Primer	Sequence (5′ to 3′)	Purpose
LP-Fw	TTGATACCAAACCATCGAACC	To genotype *prx35-1*
RP-Rv	GCAAACCACATCTCCACTTTC	
LP-Fw	AAAAAGGGCATTGCTTTTCTC	To genotype *per35-2*
RP-Rv	TGGTTGTATCGGGAAAGACAG	
BP (LBb1.3)-Fw	TGGTTCACGTAGTGGGCCATCG	To genotype both *prx35* alleles
UBQ10-Fw	CACCAGTCTAGCTCAACAGAGC	To generate the *pk7p* containing *TCTP13VENUS* and *pUB10mRFP-WAVE*
UBQ10-Rv	CGAGGGTATTGTTTTATAGAAGAAG	
03Wave 5782-Fw	CTTAATAGCGGTGGCAGCCCAGAAGGAGATATAACCATGGCGTCTGAATTGCCG	
bGH-Rv	CGAGGCGGTTCGAGAAATGG	
NLSYFP-Fw	CTGAGTTTTTCTGATTAACAGGATCCAAC	
RE9ter-Rv	TACGTAAAGCTTAGGTCGATTGATGCATGTTGTCAATCAATTGGC	
AtTCTP-Fw	CACCCAACACTCGAATCCCCACC	
AtTCTP-Rv	GGTCGCTTATTGATTGTTTCTCTCTCCG	
EF1A-Fw	AGGCTGGTATCTCCAAGGATGG	To perform RT-qPCR
EF1A-Rv	TGGCATCCATCTTGTTACAACAGC	
UBQ10-Fw	TGACAACGTGAAGGCCAAGATCC	
UBQ10-Rv	ATACCTCCACGCAGACGCAACAC	
GLV9-Fw	CGAGGCGGTTCGAGAAATGG	
GLV9-Rv	CATCAGACGATCAACCTCTTCC	

RAP2-Fw	CGGATGTGGCTCGGAACCTTTG	
RAP2-Rv	GCTGCTTCATGCTCTGCTTCTGG	
COBL9-Fw	CCTGTGCCTACTCTTCAAAGCAA	
COBL9-Rv	GTCAAGTCTCCGGGTTGTCG	
SMR7-Fw	GCCAAAACATCGATTCGGGCTTC	
SMR7-Rv	TCGCCGTGGGAGTGATACAAAT	
NRT2-Fw	AACAAGGGCTAACGTGGATG	
NRT2-Rv	CTGCTTCTCCTGCTCATTCC	
SMR5-Fw	AAACTACGACGACGGAGATACG	
SMR5-Rv	GCTACCACCGAGAAGAACAAGT	
PER35-Fw	GTTGTTGCGGCTGGAGGACCG	
PER35-Rv	GGGTGTGAGCCGCTGAAAGAGC	
F1-Fw	ACAACACTCTCTCTCTCTCGGT	To perform ChIP-qPCR; primer pair for region –34 to +79 nt of *PRX35*
R1-Rv	GTTGTGCGGTGGTTGTATCG	
F2-Fw	CCGAGATGTTGTTGTTGCGG	ChIP-qPCR; primer pair for region +487 to 644 nt of *PRX35*
R2-Rv	ACGGTCCTCCAGCCTATACA	
F3-Fw	CGCCCAAGACGTTTGACAAC	ChIP-qPCR; primer pair for region +1063–1192 nt of *PRX35*
R3-Rv	GTAGAGTTTGAGGCCCAAGC	

Table S2. Differentially expressed genes in *atx1 setm* vs wild type (Ws)

				Ws expression			*atx1 setm* - Expression		
AGI ID	P-value	Fold Change	FDR p-value	Rep 1	Rep 2	Mean	Rep 1	Rep 2	Mean
AT1G01453_1	4.22E-05	-5.526	0.0050	265	301	283	37	61	49
AT1G01580_1	1.05E-06	4.341	0.0003	1485	2032	1758.5	8928	6388	7658
AT1G01750_1	1.87E-05	-3.557	0.0026	1754	1261	1507.5	447	368	407.5
AT1G02205_1	8.01E-07	-8.260	0.0002	301	583	442	51	56	53.5
AT1G02570_1	6.99E-06	-6.610	0.0012	162	143	152.5	27	18	22.5
AT1G04150_1	6.53E-05	-6.025	0.0071	84	55	69.5	13	9	11
AT1G05020_1	6.70E-05	-5.823	0.0072	85	66	75.5	15	10	12.5
AT1G05650_1	1.39E-07	8.667	0.0000	38	63	50.5	418	450	434
AT1G07500_1	2.67E-05	6.349	0.0034	42	42	42	169	334	251.5
AT1G08090_1	6.05E-11	10.062	0.0000	468	788	628	5078	7323	6200.5
AT1G08430_1	2.03E-05	-3.601	0.0028	2233	1683	1958	690	378	534
AT1G09380_1	5.20E-06	5.698	0.0009	75	85	80	387	494	440.5
AT1G09750_1	1.75E-08	-5.043	0.0000	6104	5873	5988.5	1194	1114	1154
AT1G11600_1	2.12E-08	8.013	0.0000	73	79	76	533	644	588.5
AT1G12040_1	3.16E-08	-4.729	0.0000	13294	9914	11604	2473	2236	2354.5
AT1G12630_1	2.41E-05	7.752	0.0032	14	29	21.5	122	205	163.5
AT1G13400_1	6.34E-07	20.978	0.0002	2	2	2	32	52	42
AT1G13710_1	5.78E-06	-5.841	0.0010	430	400	415	54	81	67.5
AT1G15330_1	7.39E-07	-14.009	0.0002	157	54	105.5	10	4	7
AT1G15540_1	5.89E-14	-29.536	0.0000	596	306	451	19	10	14.5
AT1G16440_1	2.55E-05	-4.932	0.0033	379	285	332	80	51	65.5
AT1G18870_1	8.62E-06	-3.875	0.0014	1654	1816	1735	545	347	446
AT1G19900_1	2.96E-05	-3.699	0.0037	1496	1028	1262	296	348	322
AT1G21530_2	6.56E-05	-6.374	0.0071	93	59	76	16	7	11.5
AT1G21890_1	9.93E-06	-24.892	0.0016	17	38	27.5	1	1	1
AT1G23720_1	9.20E-05	-2.932	0.0093	149560	91672	120616	46856	32252	39554
AT1G24485_1	2.78E-05	-25.088	0.0035	16	16	16	0	1	0.5
AT1G25240_1	2.55E-06	-9.090	0.0005	117	78	97.5	8	12	10
AT1G26360_1	2.45E-05	-6.054	0.0032	164	170	167	23	30	26.5
AT1G26390_1	2.06E-08	14.053	0.0000	112	20	66	1105	615	860
AT1G26945_1	4.44E-05	-3.756	0.0052	892	637	764.5	213	178	195.5
AT1G27140_1	6.23E-06	-4.002	0.0011	1554	1707	1630.5	385	408	396.5
AT1G27740_1	5.87E-05	-4.397	0.0065	460	289	374.5	86	76	81
AT1G29395_1	7.52E-06	-9.578	0.0013	55	37	46	5	4	4.5
AT1G29660_1	1.15E-07	-6.903	0.0000	555	477	516	73	71	72
AT1G30170_1	1.10E-06	167.895	0.0003	0	0	0	15	25	20
AT1G30210_1	1.50E-09	-10.769	0.0000	440	555	497.5	54	38	46
AT1G30370_1	5.56E-06	-4.854	0.0010	1027	517	772	146	151	148.5
AT1G30760_1	4.18E-05	3.272	0.0050	869	912	890.5	2811	2856	2833.5
AT1G33090_1	5.16E-05	-4.785	0.0059	279	313	296	62	59	60.5

AT1G34042_1	2.57E-06	16.488	0.0005	4	5	4.5	38	104	71
AT1G34047_1	9.07E-05	11.564	0.0092	7	0	3.5	27	48	37.5
AT1G34510_1	1.31E-09	-9.811	0.0000	544	492	518	52	50	51
AT1G34540_1	8.44E-10	-15.404	0.0000	222	179	200.5	14	11	12.5
AT1G36180_1	3.21E-06	4.911	0.0006	554	477	515.5	3833	1282	2557.5
AT1G48930_1	9.25E-05	-3.044	0.0093	2818	2119	2468.5	937	640	788.5
AT1G51770_1	3.32E-05	-7.573	0.0041	58	51	54.5	10	4	7
AT1G52690_1	1.81E-11	-40.221	0.0000	185	114	149.5	6	1	3.5
AT1G52790_1	8.13E-15	-47.412	0.0000	563	247	405	11	5	8
AT1G52800_1	2.41E-06	-7.291	0.0005	242	144	193	26	24	25
AT1G52820_1	1.23E-27	-63.594	0.0000	5476	2674	4075	83	40	61.5
AT1G53130_1	8.19E-06	8.404	0.0014	56	112	84	357	1002	679.5
AT1G53680_1	1.99E-05	-5.022	0.0028	433	276	354.5	82	54	68
AT1G54890_1	2.73E-05	4.525	0.0035	305	203	254	725	1395	1060
AT1G54940_1	1.99E-05	-6.074	0.0028	237	141	189	39	21	30
AT1G54970_1	1.78E-17	-13.674	0.0000	4075	3359	3717	291	236	263.5
AT1G55650_1	2.00E-06	136.370	0.0004	0	0	0	16	17	16.5
AT1G58270_1	1.14E-09	-5.892	0.0000	5280	4532	4906	978	655	816.5
AT1G60050_1	2.42E-07	-9.539	0.0001	198	215	206.5	28	15	21.5
AT1G60190_1	1.74E-07	-6.002	0.0001	1517	979	1248	282	126	204
AT1G62500_1	2.53E-07	-4.845	0.0001	1501	1461	1481	341	259	300
AT1G63450_1	8.79E-05	-3.875	0.0090	702	513	607.5	198	109	153.5
AT1G63570_1	5.88E-07	8.725	0.0002	18	18	18	160	147	153.5
AT1G63600_1	6.11E-12	-33.429	0.0000	220	240	230	4	9	6.5
AT1G65370_1	6.89E-05	6.948	0.0074	10	4	7	59	36	47.5
AT1G65680_1	1.78E-05	5.179	0.0026	94	79	86.5	612	281	446.5
AT1G66960_1	3.40E-07	-8.659	0.0001	293	204	248.5	38	18	28
AT1G68040_1	8.14E-13	-29.433	0.0000	296	233	264.5	8	9	8.5
AT1G69240_1	1.12E-06	-4.797	0.0003	1004	901	952.5	197	187	192
AT1G69880_1	6.47E-06	4.115	0.0011	1852	2349	2100.5	6800	9907	8353.5
AT1G70460_1	9.26E-06	-3.712	0.0015	2295	1612	1953.5	626	397	511.5
AT1G71030_1	2.00E-05	-7.369	0.0028	212	104	158	13	26	19.5
AT1G72100_1	4.58E-05	-9.257	0.0054	32	28	30	2	4	3
AT1G73010_1	6.33E-07	4.061	0.0002	1339	1168	1253.5	5254	4615	4934.5
AT1G73120_1	5.02E-12	11.321	0.0000	120	132	126	1152	1590	1371
AT1G73260_1	5.95E-05	3.489	0.0065	5233	4631	4932	11641	20680	16160.5
AT1G74830_1	1.88E-06	-7.161	0.0004	271	170	220.5	35	24	29.5
AT1G77210_1	9.66E-05	23.541	0.0096	0	1	0.5	11	17	14
AT1G79860_1	1.13E-06	-7.731	0.0003	199	197	198	28	22	25
AT1G80160_1	9.38E-15	15.630	0.0000	88	57	72.5	1028	1118	1073
AT1G80320_1	1.76E-06	-13.742	0.0004	70	34	52	5	2	3.5
AT1G80580_1	7.42E-05	16.472	0.0079	1	1	1	14	21	17.5
AT2G05160_1	5.18E-05	-7.124	0.0059	577	180	378.5	80	22	51

AT2G14247_1	7.48E-06	9.171	0.0013	26	33	29.5	135	375	255
AT2G15780_1	8.08E-05	7.157	0.0084	10	6	8	40	68	54
AT2G18550_1	1.51E-09	-25.870	0.0000	81	84	82.5	3	3	3
AT2G18600_1	2.10E-07	5.466	0.0001	269	337	303	1420	1799	1609.5
AT2G18640_1	9.54E-05	-5.433	0.0095	108	80	94	17	16	16.5
AT2G18720_1	3.25E-06	7.473	0.0006	22	35	28.5	231	195	213
AT2G19500_1	2.62E-06	8.382	0.0005	17	16	16.5	184	93	138.5
AT2G19990_1	6.20E-10	8.443	0.0000	179	205	192	1243	1868	1555.5
AT2G20520_1	3.14E-11	-12.252	0.0000	603	597	600	48	47	47.5
AT2G20800_1	1.29E-05	5.987	0.0020	52	110	81	531	453	492
AT2G24255_1	2.66E-09	36.981	0.0000	2	1	1.5	72	45	58.5
AT2G24720_1	3.87E-10	9.182	0.0000	91	71	81	875	579	727
AT2G24800_1	7.34E-05	-8.776	0.0078	28	28	28	3	3	3
AT2G24980_1	7.31E-13	-7.782	0.0000	38260	28727	33493.5	4946	3421	4183.5
AT2G25150_1	1.19E-07	-7.903	0.0000	414	524	469	74	45	59.5
AT2G25160_1	1.75E-05	-3.552	0.0025	2255	2558	2406.5	800	548	674
AT2G25900_2	5.41E-05	-3.483	0.0060	2308	1235	1771.5	441	507	474
AT2G26560_1	1.13E-05	6.346	0.0018	230	66	148	1277	511	894
AT2G27535_1	1.74E-05	11.709	0.0025	7	12	9.5	50	162	106
AT2G27550_1	2.62E-05	4.662	0.0034	1208	1926	1567	4519	9549	7034
AT2G29150_1	1.70E-05	109.141	0.0025	0	0	0	18	9	13.5
AT2G29220_1	1.17E-05	9.477	0.0018	11	13	12	181	54	117.5
AT2G29250_1	3.98E-05	16.110	0.0048	3	1	2	54	14	34
AT2G29350_1	2.23E-06	9.585	0.0005	17	5	11	123	79	101
AT2G30660_1	9.66E-12	22.650	0.0000	11	23	17	443	338	390.5
AT2G30750_1	1.59E-08	8.223	0.0000	414	143	278.5	3016	1353	2184.5
AT2G30766_1	8.25E-05	8.676	0.0086	4	5	4.5	28	48	38
AT2G31141_2	5.44E-05	6.385	0.0061	47	73	60	217	516	366.5
AT2G31470_1	5.02E-05	10.026	0.0058	1	5	3	33	29	31
AT2G33020_1	4.93E-06	7.963	0.0009	13	11	12	115	74	94.5
AT2G33460_1	2.33E-06	-8.046	0.0005	285	210	247.5	46	15	30.5
AT2G33790_1	2.97E-06	-4.048	0.0006	12775	6407	9591	2078	2328	2203
AT2G34315_1	1.30E-05	19.148	0.0020	1	1	1	26	16	21
AT2G34340_1	7.04E-05	4.356	0.0075	139	135	137	432	700	566
AT2G35990_1	4.41E-05	-9.867	0.0052	32	22	27	2	3	2.5
AT2G36261_1	4.65E-10	334.725	0.0000	0	0	0	38	43	40.5
AT2G37450_2	3.49E-05	-7.572	0.0043	201	89	145	29	8	18.5
AT2G38340_1	4.20E-07	10.251	0.0001	10	11	10.5	118	95	106.5
AT2G38540_1	1.21E-05	5.399	0.0019	328	522	425	1361	3047	2204
AT2G39530_1	6.76E-05	3.270	0.0073	2135	1595	1865	4721	6739	5730
AT2G40000_1	3.94E-05	-3.445	0.0047	17356	7585	12470.5	4004	2827	3415.5
AT2G41240_1	6.62E-06	8.182	0.0012	11	22	16.5	164	111	137.5
AT2G41810_1	1.49E-10	14.953	0.0000	23	33	28	402	423	412.5

AT2G41850_1	9.25E-05	5.212	0.0093	44	20	32	186	132	159
AT2G42250_1	2.84E-12	-54.474	0.0000	140	103	121.5	1	3	2
AT2G42840_1	2.00E-06	-3.807	0.0004	12497	12109	12303	3167	3103	3135
AT2G43570_1	5.78E-05	3.478	0.0064	407	493	450	1464	1594	1529
AT2G44110_2	2.80E-05	-4.271	0.0035	897	546	721.5	214	114	164
AT2G45180_1	8.86E-05	3.808	0.0091	440	487	463.5	1164	2186	1675
AT2G45360_1	2.26E-05	5.811	0.0030	60	44	52	211	354	282.5
AT2G46860_1	2.49E-06	-5.037	0.0005	682	712	697	164	110	137
AT2G47240_1	1.32E-06	-8.003	0.0003	252	174	213	36	16	26
AT2G47780_1	2.52E-10	22.238	0.0000	9	16	12.5	218	326	272
AT3G01600_1	4.37E-13	11.507	0.0000	120	144	132	1473	1501	1487
AT3G02100_1	6.70E-14	-127.285	0.0000	199	100	149.5	2	0	1
AT3G03670_1	1.42E-06	5.186	0.0003	196	129	162.5	703	881	792
AT3G05155_1	5.18E-07	-10.355	0.0001	407	182	294.5	42	13	27.5
AT3G05950_1	5.65E-05	5.487	0.0062	28	19	23.5	125	122	123.5
AT3G07070_1	3.72E-07	-5.753	0.0001	722	597	659.5	134	90	112
AT3G07800_1	4.96E-05	3.999	0.0057	303	282	292.5	826	1384	1105
AT3G09922_1	1.64E-05	4.667	0.0024	314	372	343	1018	2024	1521
AT3G10870_1	2.52E-05	4.214	0.0033	227	296	261.5	916	1225	1070.5
AT3G11385_1	4.79E-06	-5.662	0.0009	530	348	439	99	52	75.5
AT3G12410_1	1.93E-06	10.632	0.0004	7	10	8.5	71	105	88
AT3G12540_1	6.67E-06	-6.242	0.0012	374	261	317.5	70	30	50
AT3G12900_1	1.41E-15	19.715	0.0000	55	33	44	854	798	826
AT3G13080_4	5.34E-07	-6.846	0.0001	384	326	355	52	48	50
AT3G13400_1	6.19E-05	-7.523	0.0068	39	33	36	4	5	4.5
AT3G13840_1	6.33E-09	-10.909	0.0000	308	334	321	34	24	29
AT3G13960_1	6.59E-08	-40.612	0.0000	69	27	48	2	0	1
AT3G14440_1	5.51E-07	-4.753	0.0001	1455	1079	1267	303	214	258.5
AT3G15670_1	1.28E-08	-18.016	0.0000	107	67	87	5	4	4.5
AT3G17520_1	2.47E-12	-133.541	0.0000	112	64	88	0	1	0.5
AT3G21180_1	7.16E-06	-4.786	0.0012	1000	611	805.5	220	108	164
AT3G21352_1	1.77E-06	7.240	0.0004	72	41	56.5	280	475	377.5
AT3G21520_1	1.16E-05	4.759	0.0018	108	104	106	480	496	488
AT3G21720_1	1.12E-05	5.318	0.0018	362	689	525.5	1878	3574	2726
AT3G23860_1	1.42E-05	32.613	0.0021	1	0	0.5	14	24	19
AT3G24780_1	7.82E-05	7.656	0.0082	20	3	11.5	85	78	81.5
AT3G26040_1	1.80E-05	-6.480	0.0026	360	181	270.5	59	22	40.5
AT3G26290_1	4.64E-05	-8.613	0.0054	44	39	41.5	3	6	4.5
AT3G26740_1	5.93E-08	-10.013	0.0000	650	518	584	32	76	54
AT3G27060_1	6.25E-09	5.846	0.0000	4058	4057	4057.5	18607	26683	22645
AT3G27250_1	4.33E-06	-17.462	0.0008	44	85	64.5	1	6	3.5
AT3G27630_1	1.19E-12	26.028	0.0000	26	20	23	365	751	558
AT3G27950_1	7.29E-05	4.320	0.0078	166	77	121.5	472	505	488.5

AT3G28310_1	5.07E-10	-14.596	0.0000	319	480	399.5	33	22	27.5
AT3G28320_1	2.27E-12	-11.160	0.0000	1484	2264	1874	182	154	168
AT3G28500_1	1.53E-11	31.875	0.0000	11	22	16.5	312	705	508.5
AT3G28550_1	8.71E-05	-2.953	0.0090	267070	174001	220535.5	88762	55917	72339.5
AT3G28917_1	9.77E-05	97.618	0.0097	0	0	0	7	16	11.5
AT3G29410_1	8.32E-07	-3.976	0.0002	8462	6107	7284.5	1967	1564	1765.5
AT3G30260_1	1.72E-10	-25.187	0.0000	154	133	143.5	8	3	5.5
AT3G30778_1	1.54E-05	8.673	0.0023	7	6	6.5	47	62	54.5
AT3G32030_1	2.36E-05	-6.121	0.0031	144	119	131.5	27	15	21
AT3G46190_1	7.64E-05	5.505	0.0081	20	29	24.5	160	111	135.5
AT3G47340_2	1.62E-13	10.563	0.0000	541	616	578.5	5157	6650	5903.5
AT3G48740_1	1.68E-06	-5.332	0.0004	1028	843	935.5	127	203	165
AT3G49960_1	3.00E-08	-5.182	0.0000	4833	4557	4695	794	945	869.5
AT3G50150_1	3.29E-06	-15.676	0.0006	112	39	75.5	8	1	4.5
AT3G52630_2	1.90E-05	4.896	0.0027	242	213	227.5	667	1411	1039
AT3G52780_1	1.31E-07	19.820	0.0000	4	1	2.5	54	45	49.5
AT3G52970_1	3.32E-16	-144.399	0.0000	343	154	248.5	2	1	1.5
AT3G53232_1	2.11E-05	17.215	0.0029	2	1	1.5	18	34	26
AT3G54450_1	9.55E-09	-13.724	0.0000	177	185	181	17	9	13
AT3G54580_1	6.95E-07	-4.028	0.0002	241740	161802	201771	60637	36722	48679.5
AT3G54590_1	1.66E-06	-3.711	0.0004	103744	73796	88770	27301	19044	23172.5
AT3G54820_1	1.89E-05	4.120	0.0027	375	487	431	1379	2054	1716.5
AT3G56350_1	7.87E-05	6.962	0.0082	7	6	6.5	41	47	44
AT3G58190_1	7.21E-06	5.372	0.0012	90	81	85.5	378	498	438
AT3G58270_1	4.26E-10	7.525	0.0000	210	140	175	1311	1202	1256.5
AT3G59930_1	4.35E-11	27.535	0.0000	19	7	13	223	432	327.5
AT3G60140_1	2.62E-09	16.259	0.0000	210	97	153.5	4175	799	2487
AT3G60270_1	4.13E-05	4.266	0.0049	174	208	191	635	934	784.5
AT3G60470_1	6.33E-05	-6.529	0.0069	56	46	51	9	6	7.5
AT3G61090_1	1.16E-05	16.295	0.0018	2	1	1.5	22	28	25
AT3G61930_1	4.60E-05	4.969	0.0054	52	41	46.5	229	216	222.5
AT3G61970_1	3.81E-05	-5.806	0.0046	127	131	129	21	22	21.5
AT3G62460_1	7.58E-05	6.506	0.0080	21	18	19.5	78	160	119
AT3G62680_1	8.01E-07	-3.899	0.0002	23431	20417	21924	5587	5275	5431
AT3G62760_1	1.68E-13	-12.099	0.0000	1232	1062	1147	101	83	92
AT3G63360_1	1.55E-05	4.427	0.0023	143	121	132	553	570	561.5
AT4G01630_1	4.70E-06	3.896	0.0009	608	652	630	2270	2492	2381
AT4G02270_1	8.45E-05	-3.210	0.0087	4343	3588	3965.5	982	1356	1169
AT4G05370_1	9.02E-09	28.709	0.0000	3	1	2	72	46	59
AT4G05380_1	1.02E-05	36.447	0.0016	0	1	0.5	35	11	23
AT4G08290_1	2.54E-06	-5.318	0.0005	1181	522	851.5	146	151	148.5
AT4G08300_1	1.79E-06	-3.950	0.0004	2762	2008	2385	631	530	580.5
AT4G08400_1	2.30E-09	-5.505	0.0000	6494	4854	5674	1115	876	995.5

AT4G08410_1	4.77E-10	-6.731	0.0000	2149	1643	1896	325	223	274
AT4G10500_1	4.41E-08	5.094	0.0000	500	519	509.5	3076	2068	2572
AT4G11650_1	4.21E-06	3.722	0.0008	17518	18485	18001.5	59139	70266	64702.5
AT4G11880_1	5.75E-18	-29.888	0.0000	1440	724	1082	44	25	34.5
AT4G12940_1	6.82E-08	26.600	0.0000	2	4	3	49	108	78.5
AT4G12950_1	4.90E-06	129.301	0.0009	0	0	0	13	18	15.5
AT4G13090_1	1.13E-05	-10.886	0.0018	40	31	35.5	2	4	3
AT4G13390_1	3.59E-08	-4.628	0.0000	30894	21080	25987	6200	4620	5410
AT4G13420_1	1.73E-08	-5.632	0.0000	3923	4355	4139	952	523	737.5
AT4G13720_1	9.45E-12	-15.941	0.0000	572	563	567.5	49	22	35.5
AT4G14280_1	3.52E-06	-12.197	0.0007	157	91	124	18	2	10
AT4G15370_1	2.90E-08	17.310	0.0000	5	4	4.5	74	79	76.5
AT4G15990_1	1.47E-05	-7.472	0.0022	105	66	85.5	15	7	11
AT4G16220_1	1.05E-05	-7.440	0.0017	101	80	90.5	10	13	11.5
AT4G16230_1	1.41E-06	-6.441	0.0003	350	299	324.5	56	42	49
AT4G17710_1	1.54E-13	70.227	0.0000	1	4	2.5	243	129	186
AT4G18390_1	6.53E-14	-18.235	0.0000	729	535	632	40	27	33.5
AT4G19690_2	2.48E-05	4.229	0.0033	2749	5677	4213	15802	19756	17779
AT4G19770_1	5.01E-10	-18.274	0.0000	174	149	161.5	10	7	8.5
AT4G19800_1	4.57E-05	-13.597	0.0054	27	18	22.5	3	0	1.5
AT4G21680_1	1.17E-05	6.066	0.0018	167	54	110.5	874	402	638
AT4G21750_2	3.39E-05	-4.410	0.0042	509	407	458	124	79	101.5
AT4G22080_1	1.42E-05	-4.295	0.0021	720	615	667.5	180	124	152
AT4G22105_1	1.39E-06	17.205	0.0003	4	1	2.5	35	49	42
AT4G22214_1	2.48E-10	-10.649	0.0000	719	715	717	55	74	64.5
AT4G22217_1	1.11E-08	-18.350	0.0000	123	130	126.5	4	9	6.5
AT4G22950_1	5.14E-05	-6.666	0.0059	261	112	186.5	41	13	27
AT4G22960_1	1.12E-05	4.947	0.0018	159	109	134	906	403	654.5
AT4G25310_1	2.35E-05	3.617	0.0031	441	437	439	1441	1623	1532
AT4G25330_1	8.40E-10	302.340	0.0000	0	0	0	40	34	37
AT4G25820_1	3.03E-06	-3.875	0.0006	7204	6276	6740	1490	1829	1659.5
AT4G26770_1	2.71E-08	-10.850	0.0000	275	178	226.5	24	16	20
AT4G28850_1	2.03E-11	-17.882	0.0000	530	272	401	22	20	21
AT4G29180_2	5.53E-05	-4.051	0.0061	1334	730	1032	344	152	248
AT4G30140_1	1.45E-16	12.520	0.0000	1483	1659	1571	19007	19382	19194.5
AT4G31940_1	5.60E-12	11.056	0.0000	388	728	558	7569	5048	6308.5
AT4G31970_1	4.49E-12	37.324	0.0000	40	6	23	1154	472	813
AT4G32950_1	1.38E-08	12.230	0.0000	17	30	23.5	318	260	289
AT4G33467_2	7.74E-05	-5.864	0.0081	71	79	75	14	11	12.5
AT4G33560_1	2.17E-05	4.840	0.0029	298	481	389.5	1248	2398	1823
AT4G33730_1	1.65E-05	-4.273	0.0024	726	623	674.5	147	156	151.5
AT4G34380_1	7.06E-06	-6.894	0.0012	221	119	170	29	18	23.5
AT4G34580_1	3.42E-05	-4.290	0.0042	1419	815	1117	363	147	255

AT4G35690_1	1.44E-10	-33.633	0.0000	101	145	123	3	4	3.5
AT4G37220_1	1.27E-14	-24.743	0.0000	512	505	508.5	22	18	20
AT4G38230_1	5.04E-06	-6.608	0.0009	398	197	297.5	55	31	43
AT4G39500_1	2.59E-05	18.182	0.0033	2	0	1	22	17	19.5
AT4G40090_1	1.23E-05	-3.694	0.0019	8189	7392	7790.5	1642	2361	2001.5
AT5G04120_1	1.64E-07	-6.078	0.0000	799	1069	934	182	126	154
AT5G04960_1	1.40E-06	-3.987	0.0003	5429	4874	5151.5	1187	1296	1241.5
AT5G05840_1	5.14E-05	-5.344	0.0059	154	158	156	31	26	28.5
AT5G06080_1	4.73E-08	12.609	0.0000	22	18	20	167	307	237
AT5G06500_1	9.66E-07	-24.134	0.0002	32	24	28	1	1	1
AT5G06570_1	6.38E-08	6.083	0.0000	172	140	156	832	977	904.5
AT5G06630_1	1.08E-09	-5.569	0.0000	28987	22285	25636	5350	3620	4485
AT5G06640_1	7.93E-08	-4.389	0.0000	67402	49035	58218.5	14938	10754	12846
AT5G06730_1	1.85E-05	3.432	0.0026	642	543	592.5	2253	1710	1981.5
AT5G06900_1	1.18E-18	-82.042	0.0000	753	324	538.5	6	6	6
AT5G06905_1	5.94E-23	-142.111	0.0000	1026	551	788.5	2	8	5
AT5G07050_1	3.44E-05	-16.858	0.0042	20	80	50	4	2	3
AT5G07610_1	1.56E-06	4.878	0.0003	174	152	163	779	756	767.5
AT5G08000_1	1.06E-06	5.856	0.0003	110	97	103.5	501	654	577.5
AT5G13170_1	6.55E-06	6.373	0.0012	55	98	76.5	409	552	480.5
AT5G13490_2	4.33E-06	16.311	0.0008	4	3	3.5	31	78	54.5
AT5G14490_1	5.04E-07	18.477	0.0001	1	7	4	100	56	78
AT5G14650_1	8.50E-06	3.491	0.0014	1714	1422	1568	6333	4371	5352
AT5G15380_1	3.59E-11	110.669	0.0000	1	0	0.5	85	50	67.5
AT5G15725_1	3.22E-08	-14.750	0.0000	125	124	124.5	6	10	8
AT5G16970_1	3.63E-05	3.816	0.0044	438	608	523	1655	2234	1944.5
AT5G18910_1	7.51E-06	-9.180	0.0013	56	41	48.5	6	4	5
AT5G19240_1	5.34E-05	-3.561	0.0060	3553	1455	2504	754	565	659.5
AT5G19650_1	2.14E-05	5.816	0.0029	72	75	73.5	275	533	404
AT5G19790_1	4.45E-05	-3.560	0.0052	1137	1034	1085.5	349	249	299
AT5G20045_1	3.37E-05	4.425	0.0042	212	172	192	576	1014	795
AT5G21080_1	1.33E-07	-11.858	0.0000	374	227	300.5	40	10	25
AT5G22410_1	1.00E-08	-6.061	0.0000	1401	1295	1348	234	199	216.5
AT5G22800_1	2.58E-05	4.788	0.0033	410	270	340	2535	725	1630
AT5G24770_1	1.55E-08	-5.231	0.0000	5233	5716	5474.5	1209	865	1037
AT5G26080_1	3.15E-05	-4.884	0.0039	485	328	406.5	66	90	78
AT5G33355_1	4.39E-07	29.004	0.0001	1	1	1	27	35	31
AT5G35190_1	1.06E-12	-7.871	0.0000	27009	19837	23423	3496	2296	2896
AT5G36150_1	4.83E-05	-6.012	0.0056	154	247	200.5	45	23	34
AT5G38910_1	4.41E-08	5.616	0.0000	247	277	262	1675	1240	1457.5
AT5G40780_1	7.16E-07	4.373	0.0002	895	669	782	2856	3606	3231
AT5G40860_1	9.93E-06	-6.357	0.0016	242	136	189	29	27	28
AT5G41730_1	2.60E-05	-7.598	0.0033	69	43	56	9	5	7

AT5G42510_1	3.90E-05	-3.505	0.0047	1359	1329	1344	369	374	371.5
AT5G42590_1	1.17E-05	-3.829	0.0018	1578	1276	1427	459	273	366
AT5G42600_1	8.35E-09	-23.994	0.0000	136	450	293	13	12	12.5
AT5G43120_1	2.46E-07	-52.376	0.0001	50	19	34.5	1	0	0.5
AT5G43175_1	2.42E-06	-33.733	0.0005	21	22	21.5	0	1	0.5
AT5G43230_1	6.75E-06	-5.810	0.0012	440	239	339.5	57	53	55
AT5G43330_1	9.24E-05	3.874	0.0093	1126	1633	1379.5	3507	6773	5140
AT5G43570_1	8.29E-08	6.513	0.0000	103	90	96.5	647	572	609.5
AT5G44130_1	9.20E-05	3.499	0.0093	566	477	521.5	1302	2130	1716
AT5G44260_1	1.20E-05	-5.124	0.0019	604	339	471.5	80	92	86
AT5G44440_1	3.52E-05	-3.671	0.0043	1434	1627	1530.5	360	447	403.5
AT5G45105_1	4.05E-08	12.192	0.0000	12	11	11.5	143	131	137
AT5G46950_1	5.56E-08	13.981	0.0000	6	8	7	99	95	97
AT5G48100_1	4.61E-12	-24.493	0.0000	296	197	246.5	10	9	9.5
AT5G49270_1	5.29E-05	-3.169	0.0060	2539	2312	2425.5	761	721	741
AT5G50360_1	9.38E-08	-28.897	0.0000	47	105	76	1	4	2.5
AT5G51750_1	5.67E-06	-4.575	0.0010	746	668	707	179	124	151.5
AT5G51870_3	1.55E-07	-43.074	0.0000	83	22	52.5	1	1	1
AT5G52300_1	1.31E-06	-14.657	0.0003	104	43	73.5	3	6	4.5
AT5G52390_1	5.24E-05	6.318	0.0059	45	79	62	242	515	378.5
AT5G53190_1	5.38E-05	-6.762	0.0060	90	46	68	12	7	9.5
AT5G53230_1	8.12E-13	98.865	0.0000	0	2	1	102	114	108
AT5G53240_1	4.25E-10	25.945	0.0000	4	11	7.5	173	216	194.5
AT5G54320_1	8.67E-08	24.735	0.0000	1	4	2.5	48	77	62.5
AT5G55135_1	5.68E-06	128.658	0.0010	0	0	0	22	10	16
AT5G55270_1	1.12E-11	31.074	0.0000	6	5	5.5	153	180	166.5
AT5G56080_1	1.52E-14	9.851	0.0000	691	653	672	7173	5771	6472
AT5G56450_1	2.13E-15	-225.566	0.0000	147	139	143	1	0	0.5
AT5G56460_1	6.81E-19	-96.988	0.0000	417	311	364	4	3	3.5
AT5G56600_1	8.97E-05	3.679	0.0091	246	306	276	900	1078	989
AT5G57200_1	1.57E-08	-27.740	0.0000	81	42	61.5	3	1	2
AT5G57260_1	8.28E-05	6.171	0.0086	21	14	17.5	153	62	107.5
AT5G57540_1	2.59E-05	-5.461	0.0033	258	265	261.5	40	52	46
AT5G60250_1	4.57E-23	33.025	0.0000	70	97	83.5	2837	2622	2729.5
AT5G60350_1	2.16E-05	-7.316	0.0029	234	85	159.5	27	14	20.5
AT5G61070_1	4.73E-10	16.863	0.0000	17	9	13	243	181	212
AT5G61350_1	2.30E-06	-4.950	0.0005	782	558	670	136	123	129.5
AT5G61730_1	2.35E-10	42.955	0.0000	1	3	2	120	63	91.5
AT5G61740_1	3.77E-14	45.957	0.0000	5	14	9.5	603	306	454.5
AT5G61890_1	3.39E-07	10.029	0.0001	24	9	16.5	174	140	157
AT5G62210_1	2.61E-06	-4.457	0.0005	2346	2358	2352	396	609	502.5
AT5G62330_1	6.05E-09	-12.236	0.0000	387	269	328	19	31	25
AT5G62340_1	1.44E-05	-3.935	0.0021	4020	2154	3087	586	860	723

AT5G64060_1	1.30E-10	8.895	0.0000	120	102	111	909	985	947
AT5G66400_1	2.03E-05	-12.016	0.0028	71	37	54	1	7	4
AT5G67400_1	1.86E-08	-4.946	0.0000	10253	7810	9031.5	1755	1738	1746.5

Table S3. Genes co-expressed with *PRX35*

AGI ID	Symbol	LS
AT5G04960	**PME46**	15.7814
AT5G67400	**RHS19**	13.6689
AT5G57530	XTH12	13.432
AT5G57540	XTH13	12.8397
AT1G54970	**PRP1**	12.5238
AT5G22410	RHS18	12.2277
AT4G25820	**XTH14**	11.9513
AT1G30870	PER7	11.6946
AT5G05500	MOP10	11.5762
AT3G62680	**PRP3**	11.5367
AT5G35190	EXT13	11.4972
AT4G02270	RHS13	11.4182
AT1G62980	EXPA18	11.3985
AT3G10710	RHS12	11.1221
AT4G28850	**XTH26**	11.1023
AT1G12560	EXPA7	11.0234
AT4G25790	AT4G25790	10.8457
AT4G40090	AGP3	10.7667
AT2G20520	FLA6	10.4706
AT1G34510	**PER8**	10.3126

AGI ID: Locus identifier for Arabidopsis Genome Initiative. Logic Score (LS) is a monotonic transformation (negative logit) of the mutual rank index. A higher LS indicates stronger co-expression. Bold font indicates identified differentially expressed genes in the *atx1setm* transcriptome.

Video S1 Cell lineages of the lateral root primordia (LRPs) and their 3D visualization in a time-lapse experiment. Every cell lineage of the LRPs was monitored for 37 h for Ws *TCTP1::3VENUS* (left) and 72 h for *atx1-1 TCTP1::3VENUS* (right), starting from Stage I LRP. Images were acquired every hour. The data were processed using Live Plant Cell Tracking (LiPlaCeT) Fiji plugin and then analyzed with ParaView software. Small arrows indicate cell division directions, and thick arrows show the principal growth direction within an LRP. The magnification is the same as in Fig. S5.

References

Parizot B, Roberts I, Raes J, Beeckman T, De Smet I. 2012. In silico analyses of pericycle cell populations reinforce their relation with associated vasculature in *Arabidopsis*. *Philosophical Transactions of the Royal Society B: Biological Sciences* **367**(1595): 1479-1488.

Zhang T-Q, Xu Z-G, Shang G-D, Wang J-W. 2019. A single-cell RNA sequencing profiles the developmental landscape of *Arabidopsis* root. Molecular plant **12:** 648-660.
